# Differential modulation of *C*. *elegans* motor behavior by NALCN and two-pore domain potassium channels

**DOI:** 10.1371/journal.pgen.1010126

**Published:** 2022-04-28

**Authors:** Chuanman Zhou, Qian Zhou, Xiaohui He, Yunxia He, Xiaoqin Wang, Xiaowei Zhu, Yujia Zhang, Long Ma

**Affiliations:** 1 Center for Medical Genetics, School of Life Sciences, Central South University, Changsha, Hunan, China; 2 Hunan Key Laboratory of Animal Models for Human Diseases, Central South University, Changsha, Hunan, China; 3 Hunan Key Laboratory of Medical Genetics, Central South University, Changsha, Hunan, China; 4 Hunan Key Laboratory of Molecular Precision Medicine, Central South University, Changsha, Hunan, China; Brown University, UNITED STATES

## Abstract

Two-pore domain potassium channels (K2P) are a large family of “background” channels that allow outward “leak” of potassium ions. The NALCN/UNC80/UNC79 complex is a non-selective channel that allows inward flow of sodium and other cations. It is unclear how K2Ps and NALCN differentially modulate animal behavior. Here, we found that loss of function (lf) in the *K2P* gene *twk-40* suppressed the reduced body curvatures of *C*. *elegans NALCN(lf)* mutants. *twk-40(lf)* caused a deep body curvature and extended backward locomotion, and these phenotypes appeared to be associated with neuron-specific expression of *twk-40* and distinct *twk-40* transcript isoforms. To survey the functions of other less studied K2P channels, we examined loss-of-function mutants of 13 additional *twk* genes expressed in the motor circuit and detected defective body curvature and/or locomotion in mutants of *twk-2*, *twk-17*, *twk-30*, *twk-48*, *unc-58*, and the previously reported *twk-7*. We generated presumptive gain-of-function (gf) mutations in *twk-40*, *twk-2*, *twk-7*, and *unc-58* and found that they caused paralysis. Further analyses detected variable genetic interactions between *twk-40* and other *twk* genes, an interdependence between *twk-40* and *twk-2*, and opposite behavioral effects between *NALCN* and *twk-2*, *twk-7*, or *unc-58*. Finally, we found that the hydrophobicity/hydrophilicity property of TWK-40 residue 159 could affect the channel activity. Together, our study identified *twk-40* as a novel modulator of the motor behavior, uncovered potential behavioral effects of five other *K2P* genes and suggests that NALCN and some K2Ps can oppositely affect *C*. *elegans* behavior.

## Author summary

Two-pore domain potassium channels (K2P) allow outward “leak” of potassium ions. The NALCN channel complex allows inward flow of sodium and other cations. K2Ps and NALCN are implicated in severe human diseases. Using the nematode *Caenorhabditis elegans* as a model to study the behavioral effects of K2P TWK-40 and NALCN, we found opposite effects of TWK-40 and NALCN on the body curvature. We identified five other *K2P* genes, including *twk-2*, *twk-17*, *twk-30*, *twk-48* and *unc-58*, as potential modulators of the motor behavior and confirmed the behavioral effect of the previously studied *twk-7*. These *twk* genes interacted genetically with *twk-40* in a variable manner, and *twk-2* exhibited an interdependent relationship with *twk-40*. In addition, *twk-2* and *unc-58* appeared to affect the body curvature in opposition to NALCN, and *twk-7* appeared to affect the locomotion in opposition to NALCN. Finally, we found that the chemical property of TWK-40 amino acid 159 can affect the channel activity. Together, we suggest that TWK-40 is a novel regulator of *C*. *elegans* motor behavior, five other K2Ps can potentially modulate the behavior, and some K2Ps and NALCN may oppositely affect the behavior.

## Introduction

Ion channels are pore-forming membrane proteins that allow specific ions to pass through lipid membranes via the channel pore along the concentration gradient [1]. The major channel types include chloride channels, potassium channels, sodium channels, calcium channels, proton channels and non-selective cation channels. These channels open and close in a highly coordinated manner to regulate the action potentials and set the resting membrane potentials of neurons, muscles, and other cell types. However, much remains to be understood about how these channels differentially affect the behavioral output of a neural circuit.

The two-pore domain K^+^ channels (K2P) are a large group of “leak” channels containing the characteristic four transmembrane domains and two pore-forming domains (4T2P) [2]. K2P is broadly expressed and conserved across species. The human genome encodes 15 K2Ps [3] with diverse functions. The nematode *Caenorhabditis elegans* genome encodes 47 K2Ps named TWKs (TWiK family of potassium channels) [4], most of which have unclear functions. K2Ps are voltage-insensitive in general and can be regulated by pH, temperature, mechanical stimulation, lipids and other factors [2]. K2Ps play important roles in setting the resting membrane potentials of excitable cells [5] and are implicated in the pathophysiology of nociception, neuroprotection, vascular and pulmonary hypertension, cardiac arrhythmias, depression and cancer [2].

Different from K2Ps, NALCN (Na+ leak channel, non-selective) is a non-selective cation channel that allows inward passage of Na^+^ and potentially other cations [6–8]. NALCN is conserved across species and regulated by conserved proteins [8]. In mice NALCN affects the tonic firing and excitability of substantia nigra pars reticulata neurons [9], the stable network activity within the respiratory network [7,10], and the amount of rapid eye movement sleep (REMS) and non-REMS [11]. In *Drosophila*, NALCN is required for the proper coupling of locomotion with light and dark [12] and can regulate the circadian locomotion rhythms by affecting the neural output of the circadian pacemaker [13–16]. *C*. *elegans* has two largely redundant NALCN homologs, NCA-1 and NCA-2, that together regulate the recycling of synaptic vesicles [17], synaptic transmission at neuromuscular junctions [18], neural circuit activity [19], the response to volatile anesthetics [20], locomotory patterns [21], ethanol responses [22] and developmentally timed sleep and arousal [23]. The functional conservation of NALCN is further exemplified by the findings that both *Drosophila* and *C*. *elegans* NALCN mutants are more sensitive to the volatile anesthetic halothane [20].

NALCN can be regulated by a variety of signals. In mammals, NALCN is affected by substance P via the GPCR TACR1 [24], the Src kinase [25], the M3 muscarinic receptors [26], an unidentified GPCR [27] and inhibitor GPCRs [28]. Studies in *C*. *elegans* suggest that NALCN channels are negatively regulated by dopamine through the DOP-3 receptor in command interneurons [29], are targets of Gq-Rho signaling in head acetylcholine neurons [30], and may also be modulated by the SEK-1 p38 pathway [31]. NALCN can interact with gap junctions [23,32,33].

Three proteins have been identified as specific regulators of NALCN. The ER-associated protein NLF-1 is required for proper axonal localization of *C*. *elegans* NALCN channels [34]. NALCN also physically interacts with the conserved proteins UNC79 and UNC80 (orthologs of *C*. *elegans* UNC-79 and UNC-80) [27], which are required for the channel function [8]. Loss-of-function mutations in NLF-1, UNC80 or UNC79 cause indistinguishable or highly similar phenotypes like NALCN loss-of-function mutations [15,17,18,20–22,24,25,27,35]. Mutations in NALCN and UNC80 are the cause of a group of human diseases called NALCN channelopathies [36].

We previously found that *C*. *elegans* exhibited a strong avoidance response to the odorant methyl salicylate [37]. A screen for new genes affecting this behavior identified multiple loss-of-function mutations in *unc-79* and *unc-80* [38]. To further understand the function of the NALCN channel, we performed a screen for suppressors of the “fainter” phenotype (slow locomotion and reduced body curvature) of an *unc-80* loss-of-function mutant. The screen identified two loss-of-function (lf) mutations in the *K2P* gene *twk-40*. *twk-40(lf)* strongly suppressed the “reduced body curvature” phenotype of *unc-80(lf)* and other *NALCN(lf)* mutants. In addition, *twk-40* can limit head touch-triggered backward locomotion. We further explored the functions of multiple other *twk* genes expressed in neurons of the motor circuit and identified five as potential modulators of the motor behavior. These *twk* genes genetically interacted with *twk-40* or *NALCN* to affect the motor behavior in a variable manner. Together our results suggest that *twk-40* is a novel modulator of *C*. *elegans* behavior and certain *twk* genes may affect the motor behavior in opposition to *NALCN*.

## Results

### *twk-40(lf)* mutations suppress the reduced body curvatures of *NALCN-*related loss-of-function mutants

**Fig 1:**
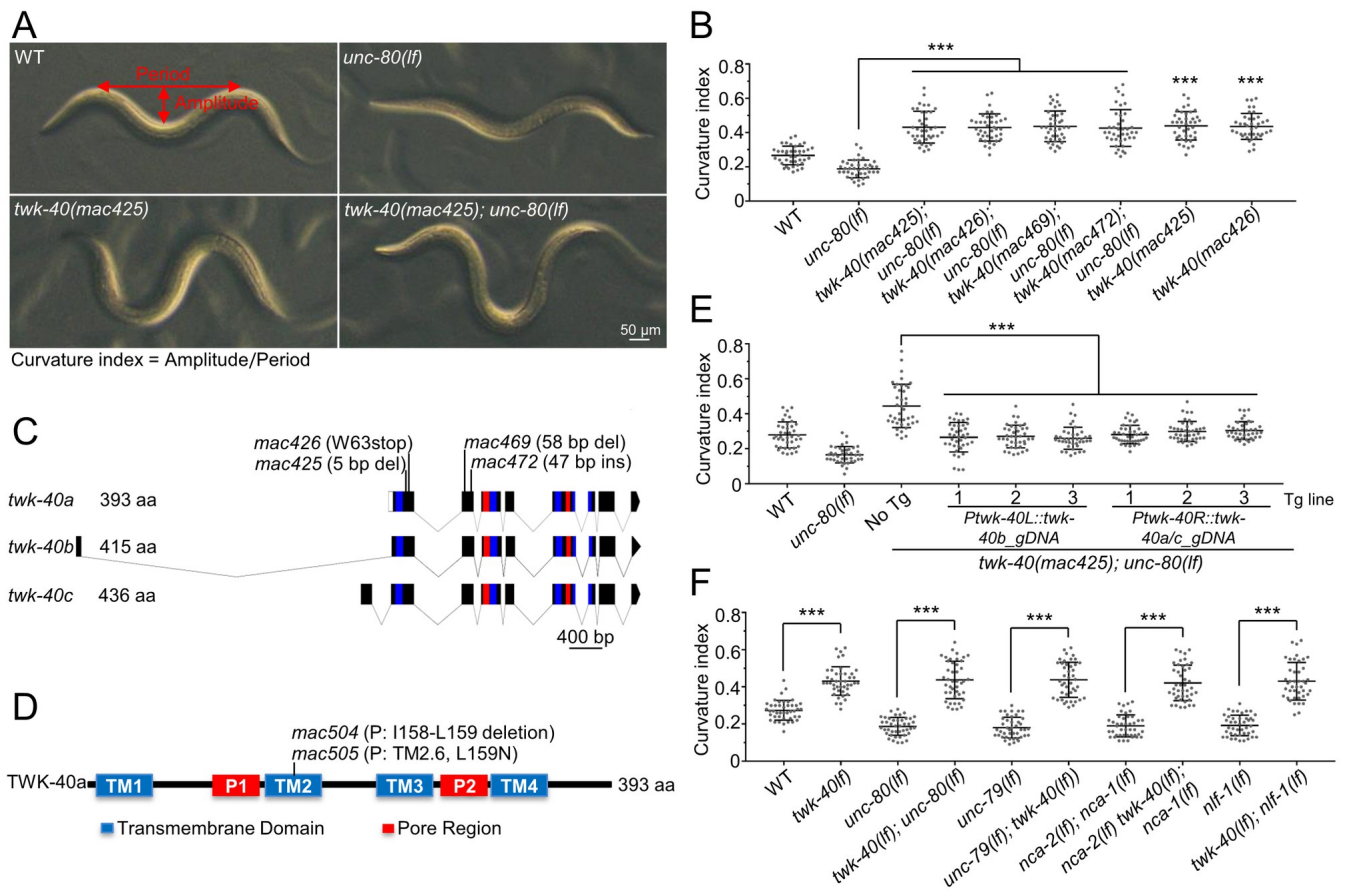
*twk-40* loss of function suppressed the reduced body curvature of *NALCN* loss-of-function mutants. (A) Representative curvatures of wildtype and mutant animals. Curvature index is the ratio of the amplitude to the period on the S-shaped body of an animal (top left panel). A low index indicates a reduced curvature, and a high index indicates a deep curvature. (B) Suppression of *unc-80(lf)* curvature defect by *twk-40(lf)* mutations. Data for *twk-40* single mutations were compared with wild type. (C) Three *twk-40* isoforms, a, b, and c are annotated at www.wormbase.org. Exons are shown as boxes and introns as lines. Exonic regions encoding the four transmembrane domains and two pore domains are marked as blue and red, respectively. Positions of loss-of-function mutations are indicated on top. (D) TWK-40a protein domains. TM: transmembrane domain. P: pore domain. The two gain-of-function mutations, *mac504* and *mac505*, affect TM2. (E) *twk-40* transgenes rescued the suppression of *unc-80(lf)* curvature defect by *twk-40(lf)*. 40 animals from each of three independent transgenic lines were quantified. (F) *twk-40(lf)* suppressed the reduced curvature of *unc-79(lf)*, *nca-2(lf); nca-1(lf)* and *nlf-1(lf)* mutants. 40 animals were quantified for each genotype. Statistics: Bonferroni multiple comparison with one-way ANOVA or two-tailed unpaired Student’s *t*-test. ***, *p* < 0.001.

We recently found that *unc-80*, *unc-79* and *NALCN* genes were required for *C*. *elegans* avoidance to the odorant methyl salicylate [38]. To identify new genes potentially interacting with *unc-80*, we performed a screen for mutations that can suppress the reduced locomotion and/or reduced body curvature of *unc-80(mac379)* mutants (Fig 1A, top right panel). *mac379* caused a G927R substitution and a W1524stop mutation in the UNC-80a protein and was characterized as a null allele of *unc-80* [38] (*unc-80(lf)* hereafter).

The screen isolated five recessive suppressors (S1 Table, top). *mac420*, *mac422* and *mac424* weakly or moderately suppressed the defective forward locomotion of *unc-80(lf)* mutants (S1A Fig). The other two suppressors, *mac425* and *mac426*, did not obviously suppress the defective basal locomotion of *unc-80(lf)* mutants or touch-triggered forward locomotion (S1A Fig), but significantly suppressed the reduced body curvature of *unc-80(lf)* mutants (Fig 1A and 1B). Using genetic analyses and/or SNP mapping, we located these mutations, except *mac424*, to chromosomes (S1 Table, top). We found that *mac425* and *mac426* probably affected a same gene on Chr. III (S1 Table, top).

We performed whole-genome sequencing on these mutants. A comparison of genes with deleterious variations on Chr. III found that *twk-40* was the only one affected in both *mac425* and *mac426* mutants (S1 Table, bottom). The analyses of other mutants are ongoing.

*twk-40* (Fig 1C and 1D) encodes a *C*. *elegans* K2P channel homologous to human KCNK9 (also known as TASK-3 or TASK3) and KCNK16 (also known as TALK-1 or TALK1) [3,39–41] (S2 Fig). *twk-40* has three annotated isoforms, a, b, and c (Fig 1C) (www.wormbase.org). The *twk-40a* isoform has eight exons and is predicted to encode a 393 aa channel shared by TWK-40b and TWK-40c isoforms (Fig 1C). *twk-40b* has an isoform-specific upstream exon and is predicted to encode a 415 aa channel (Fig 1C). *twk-40c* also has an isoform-specific upstream exon that is located at the 3’ region of the first intron of *twk-40b* (Fig 1C). *twk-40c* is predicted to encode a 436 aa channel. *mac425* caused a 5 bp deletion in exon 1 of *twk-40a*, and *mac426* caused a nonsense mutation (W63stop) in exon 1 of *twk-40a* (Fig 1C and S2 Table).

To confirm the suppressor activity of *twk-40*, we generated two novel frameshift mutations in *twk-40* using the CRISPR/Cas9 method (Fig 1C and S2 Table, *mac469* and *mac472*). Both mutations significantly suppressed the reduced curvature of *unc-80(lf)* mutants (Fig 1B). We backcrossed *twk-40(mac425)* and *twk-40(mac426)* to single mutations and found that they caused similarly deep curvature as in the double mutants (Fig 1A, bottom left panel and Fig 1B).

To verify the suppressor activity of *twk-40*, we performed transgene rescue experiments (Fig 1E). A transgene predicted to express all *twk-40* isoforms (*Ptwk-40L*::*twk-40b_gDNA*. See S3A Fig for promoter) significantly rescued the deep curvature of *twk-40(mac425); unc-80(lf)* mutants (Fig 1E). In addition, a transgene predicted to express isoforms *a* and *c* (*Ptwk-40R*::*twk-40a/c_gDNA*. See S3A Fig for promoter) exhibited similar rescuing effects (Fig 1E). Together, these results suggest that *twk-40(lf)* mutations can suppress the reduced curvature of *unc-80(lf)* mutants. To simplify our analyses, we used *twk-40(mac425)* as the reference loss-of-function allele hereafter.

Like *unc-80(lf)*, loss-of-function mutations in *unc-79*, *nca-2; nca-1* (*NALCN* null) or *nlf-1* all caused reduced curvatures. Similarly, *twk-40(lf)* significantly suppressed the reduced curvatures of these mutants (Fig 1F). Previous studies suggest that *nca-1* and *nca-2* were largely redundant in affecting *C*. *elegans* behaviors [18,20,21], while differential behavioral effects of these genes were also observed [22,30,38]. We found that *nca-1* and *nca-2* redundantly affected the curvature phenotype (S1B Fig).

### *twk-40* promoters are active in neurons

**Fig 2:**
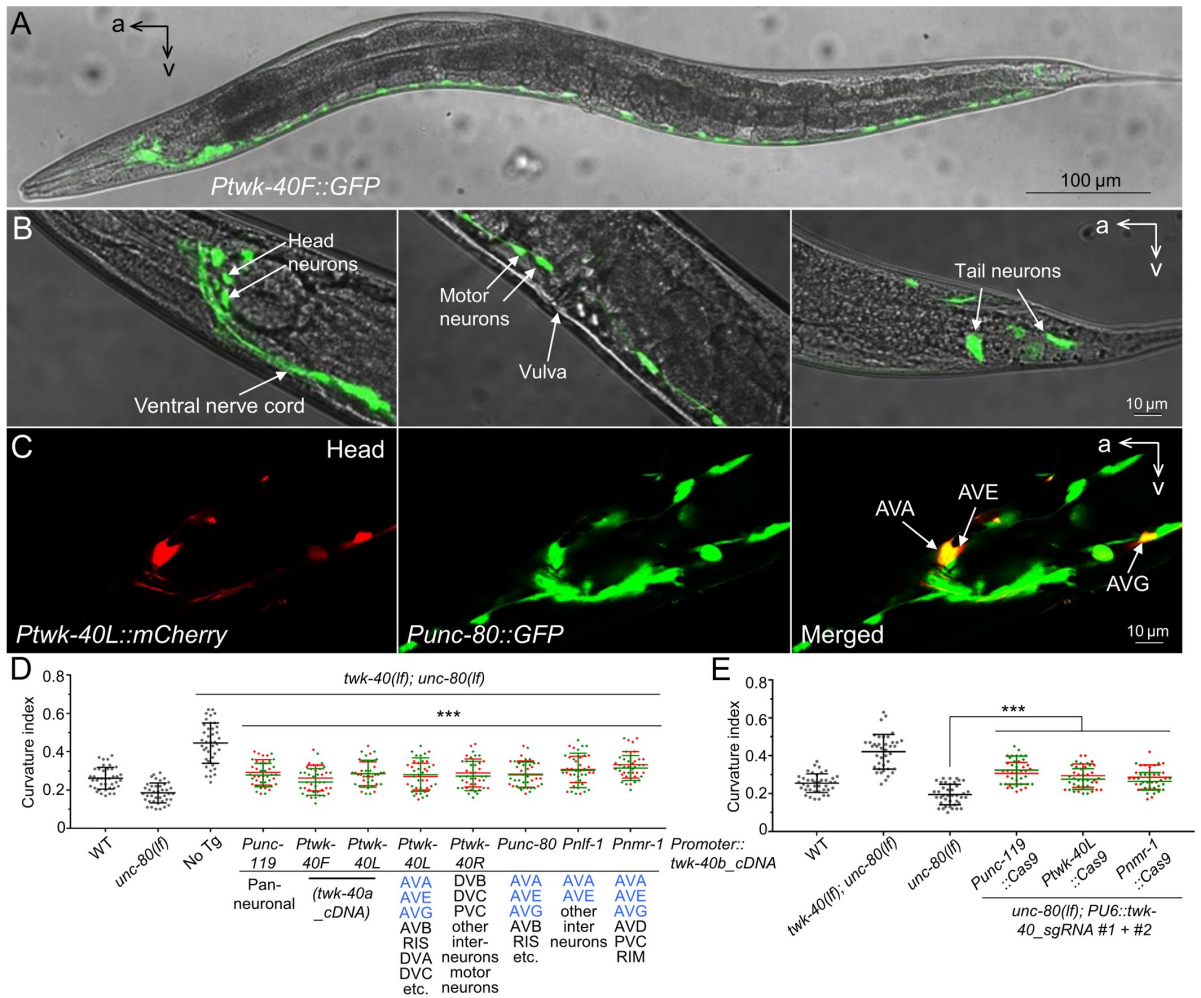
Neuronal expression of *twk-40* transgenes rescued the suppression of *unc-80(lf)* curvature by *twk-40(lf)*. (A) Picture of a transgenic adult showing representative GFP expression under control of the full-length *Ptwk-40L* promoter. (B) Some head neurons and ventral nerve cord (left panel), motor neurons (middle panel), and tail neurons (left panel) were labeled by *Ptwk-40L*::*GFP*. (C) Confocal pictures of head neurons labeled by *Ptwk-40L*::*mCherry* (left panel) and *Punc-80*::*GFP* (middle panel). The merged picture is shown on the right. AVA, AVE and AVG are indicated. a: anterior; v: ventral. (D) Neuron-specific *twk-40* transgene rescue results. Promoters are shown next to the x-axis. Neurons labeled by the promoters are listed. The asterisks above the transgenic groups indicate comparison with “No Tg”. (E) Neuron-specific knockdown of *twk-40* in *unc-80(lf)* mutants. 20 animals from each of two independent transgenic lines were quantified. Data of the two lines were shown in red and green and were used together for comparison. Statistics: Bonferroni multiple comparison with one-way ANOVA. ***, *p* < 0.001.

To identify the cells in which *twk-40* might be expressed, we generated transgenic animals expressing *GFP* driven by a 5.8 kb *twk-40* promoter (Figs 2A and S3A, *Ptwk-40F*). The transgene labeled head neurons (Fig 2B, left panel), the ventral nerve cord (Fig 2B, left panel), motor neurons (Fig 2B, central panel) and tail neurons (Fig 2B, right panel).

To investigate whether shorter *twk-40* promoters might exhibit neuronal specificity, we examined an upstream portion (S3A Fig, *Ptwk-40L*, 2.5 kb, upstream of *twk-40b* first exon) and a downstream portion (*Ptwk-40R*, 2.8 kb, upstream of *twk-40a* first exon, including the first exon of *twk-40c*) of the *Ptwk-40F* promoter. A *Ptwk-40L*::*GFP* reporter and a *Ptwk-40R*::*mCherry* reporter both labeled *C*. *elegans* head neurons, the ventral nerve cord, and tail neurons (S3B and S3C Fig). However, it appeared that *Ptwk-40R*::*mCherry* also labeled motor neurons (S3C Fig) while *Ptwk-40L*::*GFP* did not (S3B Fig).

We next crossed the two transgenes into same animals (S4 Fig). In these animals, we failed to identify head neurons obviously co-labeled by the two reporters (S4A and S4B Fig). Interestingly, an unidentified tail neuron at the position of DVA/DVB/DVC was co-labeled (S4C Fig). Consistently, multiple motor neurons were labeled by *Ptwk-40R*::*mCherry* but not by *Ptwk-40L*::*GFP* (S4D Fig).

### *twk-40* promoters and an *unc-80* promoter drive co-expression in some neurons

To examine whether *twk-40* and *unc-80* are co-expressed, we crossed a *Ptwk-40L*::*mCherry* transgene (S5 Fig) or a *Ptwk-40R*::*mCherry* transgene (S6 Fig) into animals with a *Punc-80*::*GFP* transgene [38].

In animals co-expressing *Ptwk-40L*::*mCherry* and *Punc-80*::*GFP*, the reporters co-labeled some head neurons (Figs 2C and S5) and unidentified tail neuron(s) at the position of DVA/DVB/DVC (S5 Fig). Several co-labeled neurons appeared to be AVA (Figs 2C and S5A), AVB (S5A Fig), AVE (Figs 2C and S5A), AVG (Fig 2C) and RIS (S5B Fig).

In animals co-expressing *Ptwk-40R*::*mCherry* and *Punc-80*::*GFP*, the reporters co-labeled some unidentified head neurons (S6A and S6B Fig), unidentified tail neuron(s) at the position of DVA/DVB/DVC (S6C Fig) and a subset of motor neurons (S6D Fig).

We further crossed a *Ptwk-40R*::*GFP* or a *Ptwk-40L*::*GFP* transgene into animals with a *Pnmr-1*::*mCherry* transgene. In these animals, we failed to identify head neurons co-labeled by *Ptwk-40R*::*GFP* and *Pnmr-1*::*mCherry* (S7A Fig), including the AVD and RIM neurons known to be labeled by the *Pnmr-1* promoter [42,43]. Interestingly, PVC neurons in the tail, which are major command interneurons for forward locomotion [44,45], were co-labeled by *Ptwk-40R*::*GFP* and *Pnmr-1*::*mCherry* (S7B Fig). Consistent with our earlier results (Fig 2C), *Ptwk-40L*::GFP and *Pnmr-1*::*mCherry* appeared to co-label AVA, AVE and AVG neurons (S7C Fig).

Considering that previous studies suggested the involvement of DVA and DVC neurons in locomotion [46–48], we tried to narrow down the unidentified tail neuron(s) labeled by the *Ptwk-40* promoters at the position of DVA/DVB/DVC (S4, S5 and S6 Figs). We generated transgenic animals co-expressing *Ptwk-40F*::*mCherry* and *Ptrp-4*::*GFP* or *Plim-6*^*int4*^::*GFP*. The *Ptrp-4* promoter and the *Plim-6*^*int4*^ promoter were previously shown to label DVA/DVC neurons [49,50] and DVB neurons [51], respectively. Interestingly, *Ptwk-40F*::*mCherry* co-labeled DVA/DVC with *Ptrp-4*::*GFP* (S8A Fig) and sometimes DVB with *Plim-6*^*int4*^::*GFP* (S8B Fig). We next generated transgenic animals co-expressing *Plim-6*^*int4*^::*GFP* with *Ptwk-40L*::*mCherry* or *Ptwk-40R*::*mCherry*. Here we found that *Ptwk-40L*::*mCherry* labeled DVA/DVC but not DVB (S8C Fig), while *Ptwk-40R*::*mCherry* labeled DVB/DVC but not DVA (S8D Fig).

### Neuronal *twk-40* is important for the expression of *unc-80(lf)* curvature phenotype

To examine the neuron-specific activities of *twk-40*, we used different promoters to drive the expression of a *twk-40b* cDNA transgene in *twk-40(lf); unc-80(lf)* double mutants (Fig 2D). The *twk-40b* transgene driven by a *Punc-119* pan-neuronal promoter [52] and five other promoters, including *Ptwk-40L* (S3A Fig), *Ptwk-40R* (S3A Fig), *Punc-80* [38], *Pnlf-1* [34], and *Pnmr-1* [42], all exhibited significant rescuing effects (Fig 2D).

To understand whether TWK-40 isoforms might differ in channel activity, we tested a *twk-40a* cDNA transgene driven by the *Ptwk-40F* promoter, which is predicted to also express *twk-40b* and *twk-40c*, or by the *Ptwk-40L* promoter. These transgenes exhibited similar rescuing effects compared to the *twk-40b* cDNA transgenes (Fig 2D). Reasoning that *twk-40a* encodes the common channel region of all three TWK-40 isoforms, we chose *twk-40a* cDNA as the representative isoform in the following transgenic experiments.

The effect of neuronal *twk-40* on the curvature of *unc-80(lf)* mutants was also confirmed by neuron-specific knockdown experiments. Using a *Punc-119*::*Cas9* transgene and two *twk-40*-specific sgRNAs to disrupt *twk-40* coding sequences, we could suppress the reduced curvature of *unc-80(lf)* mutants to a deeper one (Fig 2E). *Cas9* driven by *Ptwk-40L* or *Pnmr-1* exhibited similar effects (Fig 2E).

### Differential effects of *twk-40* promoters on the body curvature

Earlier transgene rescue experiments were primarily performed in *twk-40(lf); unc-80(lf)* double mutants (Figs 1E and 2D). To simplify the analyses of *twk-40*, we performed more rescue experiments in *twk-40(lf)* single mutants using the *twk-40a* cDNA transgene driven by different promoters.

**Fig 3:**
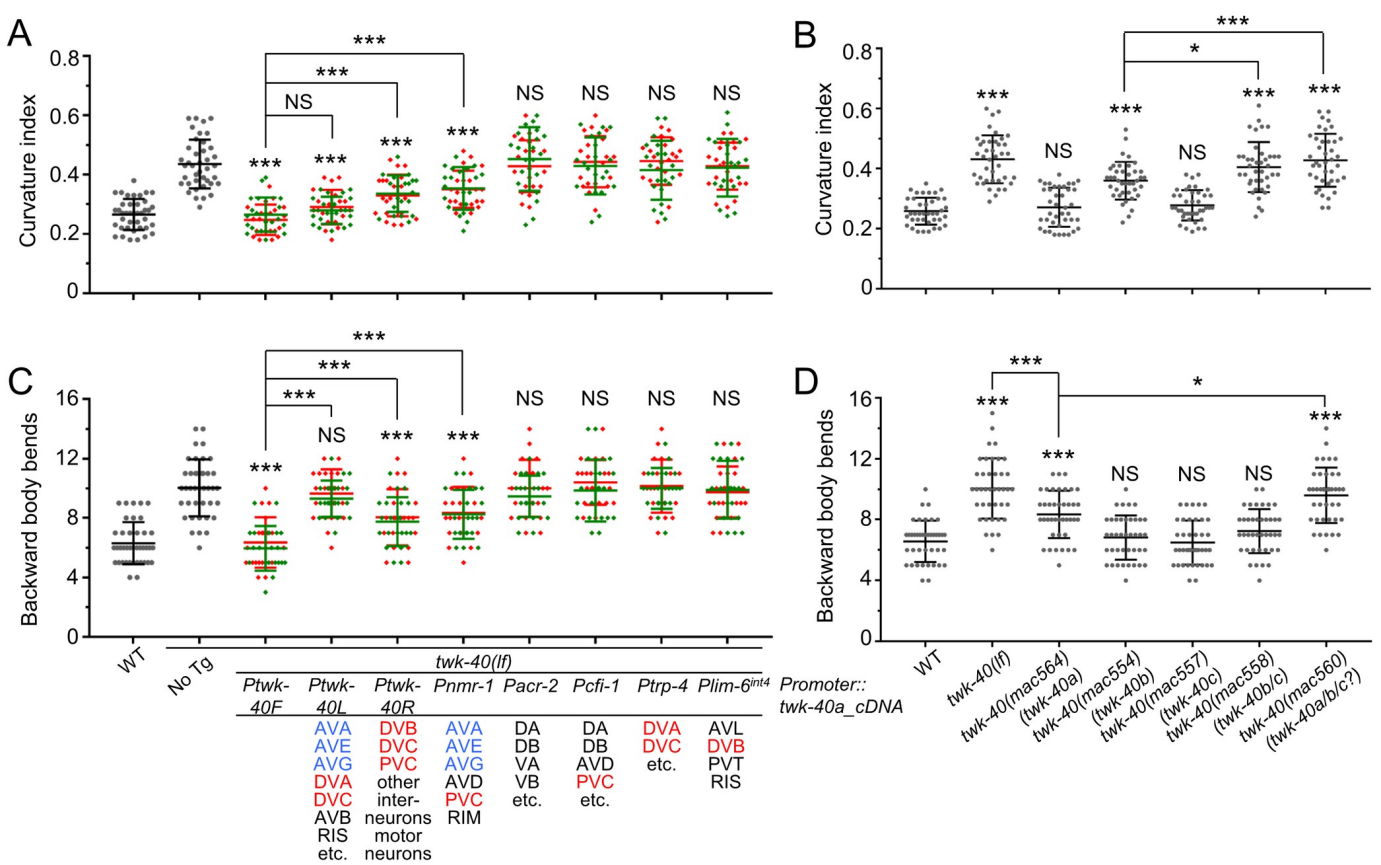
Different effects of *twk-40* transgenes and isoform-specific mutations on the curvature and backward locomotion. (A) Curvature indices of *twk-40(lf)* mutants expressing *twk-40a* cDNA transgenes driven by different promoters. The genotypes are shown at the respective positions in (C). (B) Curvature indices of presumptive *twk*-*40* isoform-specific mutants. The genotypes are shown at the respective positions in (D). (C) Touch-triggered backward locomotion of *twk-40(lf)* mutants expressing different transgenes. (D) Touch-triggered backward locomotion of presumptive *twk*-*40* isoform-specific mutants. Except otherwise specified, the asterisks and “NS” above each dataset in (A) and (C) indicate comparisons with “No Tg”, and those in (B) and (D) indicate comparisons with WT. For (A) and (C), 20 animals from each of two transgenic lines were quantified. Data of the two lines were shown in red and green and were used together for comparison. For (B) and (D), 40 animals were quantified for each genotype. Statistics: Bonferroni multiple comparison with one-way ANOVA or two-tailed unpaired Student’s *t*-test. *, *p* < 0.05; ***, *p* < 0.001. NS, not significant.

The first finding was that *Ptwk-40F* rescued the deep curvature of *twk-40(lf)* mutants to the wildtype level (Fig 3A). This was different from the partial effect of this promoter on *twk-40(lf); unc-80(lf)* double mutants, in which the deep curvature was rescued to the wildtype level but not further to the *unc-80(lf)* level (Fig 2D).

*Ptwk-40L* also rescued the deep curvature of *twk-40(lf)* mutants close to the wildtype level (Fig 3A). However, *Ptwk-40R* exhibited a weaker rescuing effect, which was like *Pnmr-1* (Fig 3A).

To examine whether other neurons in which *twk-40* promoters were active are involved in the curvature phenotype, we performed transgene rescue experiments using a *Pacr-2* promoter (active in excitatory cholinergic motor neurons) [53], a *Pcfi-1* promoter (active in PVC neurons) [54], the *Ptrp-4* promoter and the *Plim-6*^*int4*^ promoter. However, none of these promoters exhibited an obvious rescuing effect on the curvature phenotype of *twk-40(lf)* mutants (Fig 3A).

### Differential effects of *twk-40* transcript isoforms on the body curvature

To examine how *twk-40* isoforms affect the curvature, we used the CRISPR/Cas9 method to generate deleterious mutations only affecting the *twk-40b*-specific exon, the *twk-40c*-specific exon, or both (S9 Fig), postulating that such mutations might reveal isoform-specific functions.

We obtained two independent mutants of each category (*twk-40b*, *twk-40c*, or both) (S9A Fig). Mutant strains of the same category exhibited indistinguishable phenotypes, and we quantified the curvature of one strain for each category.

*twk-40(mac554)* (1 bp deletion in *twk-40b* exon 1, S9 Fig) mutants exhibited a curvature deeper than wildtype animals but not as deep as *twk-40(lf)* mutants (Fig 3B). However, *twk-40(mac557)* (61 bp deletion in *twk-40c* exon 1, S9 Fig) mutants exhibited a wildtype-like curvature (Fig 3B). Interestingly, *twk-40(mac558)* mutants (1 bp deletion in *twk-40b* exon 1 and 10 bp deletion in *twk-40c* exon 1, S9 Fig) exhibited a curvature slightly deeper than *twk-40(mac554)* mutants.

While generating *twk-40(mac558)*-like mutations, we isolated *twk-40(mac560)* (2987 bp deletion spanning the start codons of *twk-40b* and *twk-40c*) (S9 Fig). *mac560* might also affect *twk-40a* expression if the deletion disrupted *twk-40a* endogenous promoter. *twk-40(mac560)* mutants exhibited a deep curvature like *twk-40(lf)* mutants (Fig 3B).

So far, these isoform-specific mutant strains were not sufficient for analyzing the effect of *twk-40a*. To address this question, we used the CRISPR/Cas9 method to mutate the start codon of *twk-40a* to a codon for isoleucine (S9 Fig, *mac564*), hoping that this mutation would specifically disrupt TWK-40a expression. The lack of an obvious curvature defect in *twk-40(mac564)* mutants (Fig 3B) suggests that this mutation did not significantly disrupt total TWK-40 channel activity, and that *twk-40a* was not critically involved in the curvature phenotype if the mutation specifically disrupted TWK-40a expression.

### *twk-40* affects touch-triggered backward locomotion

To identify other motor defects caused by *twk-40(lf)*, we quantified touch-triggered forward (body bends/30 sec) or backward locomotion (body bends until stop). We found that tail touch triggered a slightly slower forward locomotion in *twk-40(lf)* mutants (Table 1, S1 Movie for wild type and S2 Movie for *twk-40(lf)* mutants). However, head touch triggered significantly extended backward locomotion in *twk-40(lf)* mutants (6.4 ± 1.5 body bends for wild type, 10.1 ± 2.4 for *twk-40(lf)*) (Table 1, S3 Movie for wild type and S4 Movie for *twk-40(lf)* mutants).

**Table 1:** Quantification and description of behavioral phenotypes of *twk* mutants.

Genotype	Curvature index	Forward body bends/30 sec	Backward body bends	Notes
WT	0.28 ± 0.07	30.0 ± 3.5	6.4 ± 1.5	
*twk-40(lf)*	0.47 ± 0.11 ***	28.2 ± 2.8 *	10.1 ± 2.4 ***	Frequent backward locomotion
*twk-2(lf)*	0.36 ± 0.08 ***	29.5 ± 2.7	7.5 ± 1.8 *	Frequent backward locomotion
*twk-7(lf)*	0.26 ± 0.06	36.7 ± 2.8 ***	7.4 ± 1.6	
*twk-17(lf)*	0.29 ± 0.07	25.2 ± 2.5 ***	8.7 ± 1.9 ***	Longer wavelength
*twk-30(lf)*	0.27 ± 0.06	31.3 ± 2.7	10.5 ± 1.7 ***	
*twk-48(lf)*	See notes	26.8 ± 2.3 ***	5.3 ± 1.3 *	Frequent forward deep bending, brief forward coiling after backward locomotion, irregular curvature
*unc-58(lf)*	0.46 ± 0.11 ***	23.4 ± 3.1 ***	5.3 ± 1.5 *	
*twk-2(lf); twk-40(lf)*	0.38 ± 0.10 ***	29.4 ± 3.0	10.8 ± 2.3	
*twk-7(lf) twk-40(lf)*	0.37 ± 0.08 ***	32.2 ± 2.5 ***	10.7 ± 2.1	
*twk-40(lf); twk-17(lf)*	See notes	15.6 ± 2.2 ***	10.9 ± 2.1	Very deep curvature
*twk-30(lf); twk-40(lf)*	0.45 ± 0.09	27.4 ± 2.8	10.5 ± 2.4	
*twk-48(lf) twk-40(lf)*	See notes	Delta turn/Coiler	Coiler	Very deep curvature, frequently coil
*twk-40(lf); unc-58(lf)*	See notes	13.6 ± 1.8 ***	Coiler	Very deep curvature
*twk-40(mac505gf)*	See notes	0	0	Paralyzed, flaccid
*twk-2(gf)*	See notes	0	0	Paralyzed, flaccid
*twk-2(lf); twk-40(mac505gf)*	0.27 ± 0.07	14.9 ± 2.6 ***	2.3 ± 0.9 ***	Slightly flaccid
*twk-2(gf); twk-40(lf)*	See notes	1.3 ± 1.1 ***	10.3 ± 2.8 ***	Infrequent forward locomotion with normal curvature, frequent unprovoked backward locomotion with deep curvature
*twk-40(mac504gf)*	0.15 ± 0.05	8.2 ± 4.3	3.2 ± 1.8	
*twk-2(lf); twk-40(mac504gf)*	0.35 ± 0.09 ***	27.6 ± 2.3 ***	9.5 ± 2.0 ***	

For single mutants, comparisons were made with wild type. For double loss-of-function mutants, comparisons were made with *twk-40(lf)* mutants. For *twk-2(lf); twk-40(mac505gf)*, *twk-2(gf); twk-40(lf)* or *twk-2(lf); twk-40(mac504gf)* double mutants, comparisons were made with *twk-40(mac505gf)*, *twk-2(gf)* or *twk-40(mac504gf)* single mutants, respectively. 40 animals were measured for each data. Statistics: Bonferroni multiple comparison with one-way ANOVA or two-tailed unpaired Student’s *t*-test.

*, *p* < 0.05 ***, *p* < 0.001. Phenotypes that were difficult to quantify were described.

The extended backward locomotion of *twk-40(lf)* mutants was fully rescued by *twk-40a* cDNA transgenes driven by *Ptwk-40F*, and partially by *Ptwk-40R* or *Pnmr-1* (Fig 3C). However, *Ptwk-40L* did not exhibit an obvious rescuing effect, and neither did *Pacr-2*, *Pcfi-1*, *Ptrp-4* or *Plim-6*^*int4*^ (Fig 3C).

Interestingly, *twk-40(mac564)* mutants, presumptively *twk-40a*-specific, exhibited obviously extended backward locomotion, though it was weaker than *twk-40(lf)* mutants (Fig 3D). However, *twk-40b*-specific *mac554* mutants, *twk-40c*-specific *mac557* mutants and *twk-40b/c*-specific *mac558* mutants all exhibited backward locomotion not significantly different from wildtype animals (Fig 3D). Only *twk-40(mac560)* mutants, which presumably disrupted the expression of all three *twk-40* isoforms, exhibited extended backward locomotion like *twk-40(lf)* (Fig 3D).

### More *twk* genes affect *C*. *elegans* motor behavior

47 *twk* genes were annotated in the *C*. *elegans* genome (S3 Table) [4] (www.wormbase.org), the functions of most of which were unclear. The deep body curvature and extended backward locomotion of *twk-40(lf)* mutants prompted us to investigate whether other less studied *twk* genes might also affect *C*. *elegans* motor behavior.

To simplify our analyses, we searched the CeNGEN database (www.cengen.org) [55] for *twk* genes with obvious expression in neurons of the motor circuit. The motor circuit primarily includes backward premotor interneurons AVA/AVD/AVE, forward premotor interneurons AVB/PVC, excitatory backward motor neurons DA/VA, excitatory forward motor neurons DB/VB, and inhibitory motor neurons DD/VD [44,45]. The search identified 14 such *twk* genes, including *twk-40* (S3 Table, genes labeled in red and blue). Though these data probably provided only partial pictures about the *in vivo* expression of *twk* genes, we postulated that they would be useful for prioritizing our analyses.

To investigate whether these motor circuit-expressed *twk* genes might affect the locomotion, we generated two independent presumptive loss-of-function mutations in each gene using the CRISPR/Cas9 method. We also generated a mutation in *twk-3*, which was included as a negative control because it was not obviously expressed in neurons of the motor circuit (S3 and S4 Tables). Comparison of the two loss-of-function mutants of each *twk* gene suggested that they exhibited indistinguishable or very similar phenotypes. Hence, we chose one mutant of each gene for further analyses (S4 Table, mutations in bold).

We found that six *twk* mutants, including *twk-2*, *twk-7*, *twk-17*, *twk-30*, *twk-48*, and *unc-58*, exhibited distinct changes in body curvature and/or touch-triggered locomotion (Table 1). Their phenotypes were briefly described as follows.

*twk-2(lf)* mutants exhibited a moderately deep curvature (S5 Movie) and a weakly extended backward locomotion. They also frequently switched to backward locomotion without being provoked.

*twk-7(lf)* mutants exhibited hyperactive forward locomotion (Table 1), consistent with previous findings [56,57].

*twk-17(lf)* mutants (S6 Movie) exhibited moderately slower forward locomotion, moderately extended backward locomotion, and a longer body wavelength.

*twk-30(lf)* mutants (S7 Movie) exhibited extended backward locomotion like *twk-40(lf)* mutants.

*twk-48(lf)* mutants exhibited an irregular curvature (S8 Movie). These mutants also frequently made deep forward turns, exhibited brief forward coiling upon completing backward locomotion, and exhibited weakly reduced forward and backward locomotion (Table 1).

*unc-58(lf)* mutants exhibited a deep curvature (S9 Movie) like *twk-40(lf)* mutants. These mutants also exhibited reduced forward and backward locomotion (Table 1). Previous studies found that gain-of-function mutations in *unc-58* caused hyper-activation of body-wall muscles and dramatically perturbed locomotion [58,59].

To confirm that the observed phenotypes of these mutants were caused by loss-of-function mutations, we performed transgene rescue experiments. Indeed, the obvious phenotype of each mutant could be rescued by its respective wildtype transgene under control of the *unc-119* promoter (S5 Table).

We failed to observe obvious behavioral changes in eight other *twk(lf)* mutants, including the *twk-3(lf)* mutant that was treated as a negative control (S6 Table). These *twk* mutations did not obviously enhance or suppress the curvature or backward locomotion of *twk-40(lf)* mutants (S6 Table).

### Variable genetic interactions between *twk* genes

To understand how the *twk* genes with obvious behavioral effects interact genetically, we generated double mutants between *twk-40(lf)* and *twk-2(lf)*, *twk-7(lf)*, *twk-17(lf)*, *twk-30(lf)*, *twk-48(lf)* or *unc-58(lf)* (Table 1). Their phenotypes were summarized as follows.

*twk-2(lf)* or *twk-7(lf)* suppressed the deep curvature of *twk-40(lf)* mutants (S10 and S11 Movies), *twk-30(lf)* had no effect, and *twk-17(lf)* (S12 Movie), *twk-48(lf)* or *unc-58(lf)* enhanced.

*twk-2(lf)* or *twk-30(lf)* had no effect on the slightly reduced forward locomotion of *twk-40(lf)* mutants, *twk-7(lf)* improved it, and *twk-17(lf)* or *unc-58(lf)* enhanced it.

However, the extended backward locomotion of *twk-40(lf)* mutants was not obviously affected by other *twk(lf)* mutations (Table 1. See below for *twk-48* and *unc-58*), suggesting a dominant effect of *twk-40* on this behavior.

Interestingly, *twk-48(lf); twk-40(lf)* double mutants tended to coil or make delta turns upon head or tail touch (Table 1, S13 and S14 Movies). Coiling was also induced in *twk-40(lf); unc-58(lf)* double mutants by head touch but not by tail touch (Table 1, S15 and S16 Movies).

### Presumptive gain-of-function mutations in *twk* genes can cause strong paralysis

The variable phenotypes of *twk(lf)* mutants raised a question as to how gain-of-function mutations in these genes might affect the behavior. To examine this, we used the CRISPR/Cas9 method to generate mutations that changed a residue located in the 2^nd^ transmembrane domain inner helix of the channels (named TM2.6) to asparagine (N). A recent study found that similar substitutions can generate hyperactive K2P channels in vertebrates and *C*. *elegans* [60]. We were able to obtain such mutations for TWK-40a (Figs 1D, S2 and S10A, S2 Table), TWK-2, TWK-7, TWK-43, and UNC-58 channels (S4 Table and S10A Fig).

**Fig 4:**
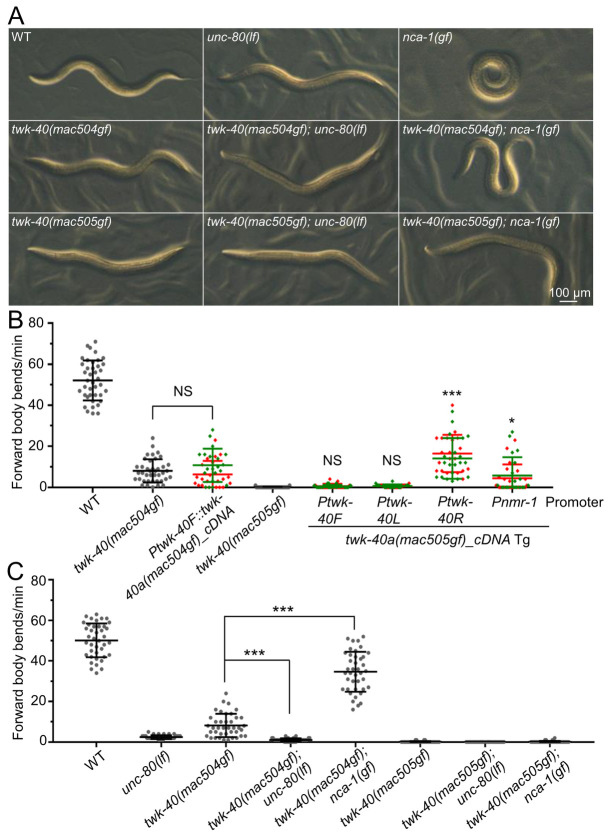
Analyses of *twk-40* gain-of-function mutations. (A) Morphologies of wild type and mutants with indicated genotypes. (B) *twk-40(gf)* transgenes phenocopied the defective locomotion of *twk-40(gf)* mutants. Except otherwise specified, the asterisks and “NS” above the transgenic groups in (B) indicate comparisons with *twk-40(mac505gf)*. 20 animals from each of two transgenic lines were quantified. Data of the two lines were shown in red and green and were used together for comparison. (C) Locomotion of animals with indicated genotypes. 40 animals were quantified for each genotype. Note: the locomotion of *nca-1(gf)* mutants was not quantified because these animals frequently coil and initiate backward locomotion during the 1-min measurement, which made it difficult to interpret whether their locomotion was locomotion only or a combination of locomotion and other disruptions. Statistics: Bonferroni multiple comparison with one-way ANOVA or two-tailed unpaired Student’s *t*-test. *, *p* < 0.05; ***, *p* < 0.001. NS, not significant.

For TWK-40a, the L159N (TM2.6) mutation (*mac505gf*) (Fig 1D and S2 Table) caused a strong paralysis (Figs 4A and S10B, bottom left panel). The paralysis phenotype was fully phenocopied by *twk-40a(mac505gf)_cDNA* transgenes driven by *Ptwk-40F* and *Ptwk-40L*, and partially by *Ptwk-40R* and *Pnmr-1* (Fig 4B). Crossing *twk-40(mac505gf)* with *unc-80(lf)* (Fig 4A, top central panel) resulted in a similar paralysis (Fig 4A, bottom central panel and Fig 4C). Interestingly, the coiler phenotype of *nca-1(gf)* mutants (Fig 4A, top right panel) was suppressed to paralysis by *twk-40(mac505gf)* (Fig 4A, bottom right panel and Fig 4C).

Among the other four TM2.6>N mutations, TWK-2a(I208N), TWK-7(L309N) and UNC-58a(F332N) all caused strong paralysis (S7 Table and S10B Fig, referred as gf hereafter). The body shapes of *twk-2(gf)* and *twk-7(gf)* mutants were flaccid, like *twk-40(mac505gf)* mutants. The body shape of *unc-58(gf)* mutants was circled (S10B Fig) or in some cases curved as previously reported [60]. We failed to observe obviously abnormal behaviors in TWK-43a(M208N) mutants (S10A Fig, S4 and S7 Tables). The underlying mechanism was unclear, and it may be worth considering the possibilities that the mutation affected the expression, folding, transportation and/or degradation of TWK-43.

### *twk-40* and *twk-2* may be partially interdependent

To understand whether the paralysis phenotype of *twk-40(gf)* mutants might be affected by other *twk* genes, we crossed *twk-40(mac505gf)* with loss-of-function mutations in *twk-2*, *twk-7*, *twk-17*, and *unc-58*. Interestingly, *twk-2(lf)* partially suppressed the paralysis of *twk-40(mac505gf)* mutants to slow locomotion (Tables 1 and S7) (S17 and S18 Movies), while *twk-7(lf)*, *twk-17(lf)* or *unc-58(lf)* failed to do so (S7 Table). The interaction between *twk-2* and *twk-40* was reciprocal, as *twk-40(lf)* also suppressed the paralysis of *twk-2(gf)* mutants to slow locomotion (Tables 1 and S7) (S19 and S20 Movies). However, *twk-40(lf)* did not obviously affect the paralysis of *twk-7(gf)* or *unc-58(gf)* mutants (S7 Table). Therefore, *twk-40* and *twk-2* might specifically depend on each other for full activities.

To examine whether *twk-2* and *twk-40* are expressed in same neurons, we generated transgenic animals co-expressing *Ptwk-40F*::*GFP* and *Ptwk-2*::*mCherry* (*Ptwk-2* is a 3.1 kb *twk-2* promoter upstream of the start codon). In these animals, we observed co-expression of the reporters in AVA and AVE neurons (S11A Fig), unidentified tail neuron(s) at the position of DVA/DVB/DVC (S11B Fig) and a subset of motor neurons (S11C Fig).

### Differential effects of *twk* genes on *NALCN* mutant phenotypes

To investigate how these newly analyzed *twk* genes might interact genetically with *NALCN-*related genes, we crossed *twk(lf)* mutations with *unc-80(lf)* and *twk(gf)* mutations with *nca-1(gf)*.

We found that all *twk(lf)* mutations failed to improve the slow locomotion of *unc-80(lf)* mutants except *twk-7(lf)*, which did it weakly (Table 2). Interestingly, *twk-2(lf)* or *unc-58(lf)*, like *twk-40(lf)*, suppressed the reduced curvature of *unc-80(lf)* mutants to deeper ones, while *twk-7(lf)* or *twk-17(lf)* failed to do so (Table 2).

**Table 2:** Genetic interactions between *twk* and *NALCN* mutations.

Genotype	Phenotype	
	**Curvature index**	**Forward body bends/30 sec**
*unc-80(lf)*	0.16 ± 0.05	2.5 ± 1.4
*twk-40(lf); unc-80(lf)*	0.46 ± 0.09 ***	3.5 ± 2.2
*twk-2(lf); unc-80(lf)*	0.35 ± 0.07 ***	3.9 ± 2.7
*twk-7(lf); unc-80(lf)*	0.17 ± 0.05	9.9 ± 7.8 ***
*unc-80(lf); twk-17(lf)*	0.19 ± 0.05	2.4 ± 1.2
*unc-80(lf); unc-58(lf)*	0.38 ± 0.07 ***	3.5 ± 1.6
*nca-1(gf)*	Coiler	
*twk-40(mac505gf); nca-1(gf)*	Paralysis	
*twk-2(gf); nca-1(gf)*	Paralysis	
*twk-7(gf); nca-1(gf)*	Paralysis	
*nca-1(gf); unc-58(gf)*	Circled paralysis	

40 animals were measured for each data. Statistics: Bonferroni multiple comparison with one-way ANOVA.

***, *p* < 0.001. Comparisons were made with *unc-80(lf)*.

Like *twk-40(mac505gf)*, *twk-2(gf)*, *twk-7(gf)* and *unc-58(gf)* all suppressed the coiler phenotype of *nca-1(gf)* mutants to paralysis phenotypes comparable with those of the individual *twk(gf)* mutants (Table 2 and S10B Fig).

### A novel form of TWK-40 gain of function

In generating the *twk-40(mac505gf)* mutation, we obtained an in-frame deletion of I158 and L159 in TWK-40a (Fig 1D and S2 Table, *mac504*). *twk-40(mac504)* mutants were sluggish and moderately flaccid (Fig 4A, middle left panel and Fig 4B). A *twk-40a(mac504)* cDNA transgene driven by *Ptwk-40F* caused a locomotion defect phenocopying that of *twk-40(mac504)* mutants (Fig 4B). Hence, *mac504* might cause a moderate gain of function in TWK-40.

We found that *twk-40(mac504gf)* and *unc-80(lf)* together caused a strong paralysis (Fig 4A, middle central panel, and Fig 4C). Interestingly, *twk-40(mac504gf)* suppressed the coiler phenotype of *nca-1(gf)* mutants to a non-coiler phenotype with very deep curvature (Fig 4A, middle right panel) and these animals exhibited a moderate locomotion (Fig 4C). Like *twk-40(mac505gf)*, the reduced curvature and defective locomotion of *twk-40(mac504gf)* mutants were also suppressed by *twk-2(lf)* (Table 1).

### TWK-40a activity might be affected by chemical properties of residue 159 and extracellular K^+^ concentration

It is intriguing that the deletion of I158 and L159 would confer a hyperactivity in TWK-40a. We examined whether deletion of either residue alone would affect the channel activity using transgene phenocopy experiments.

**Fig 5:**
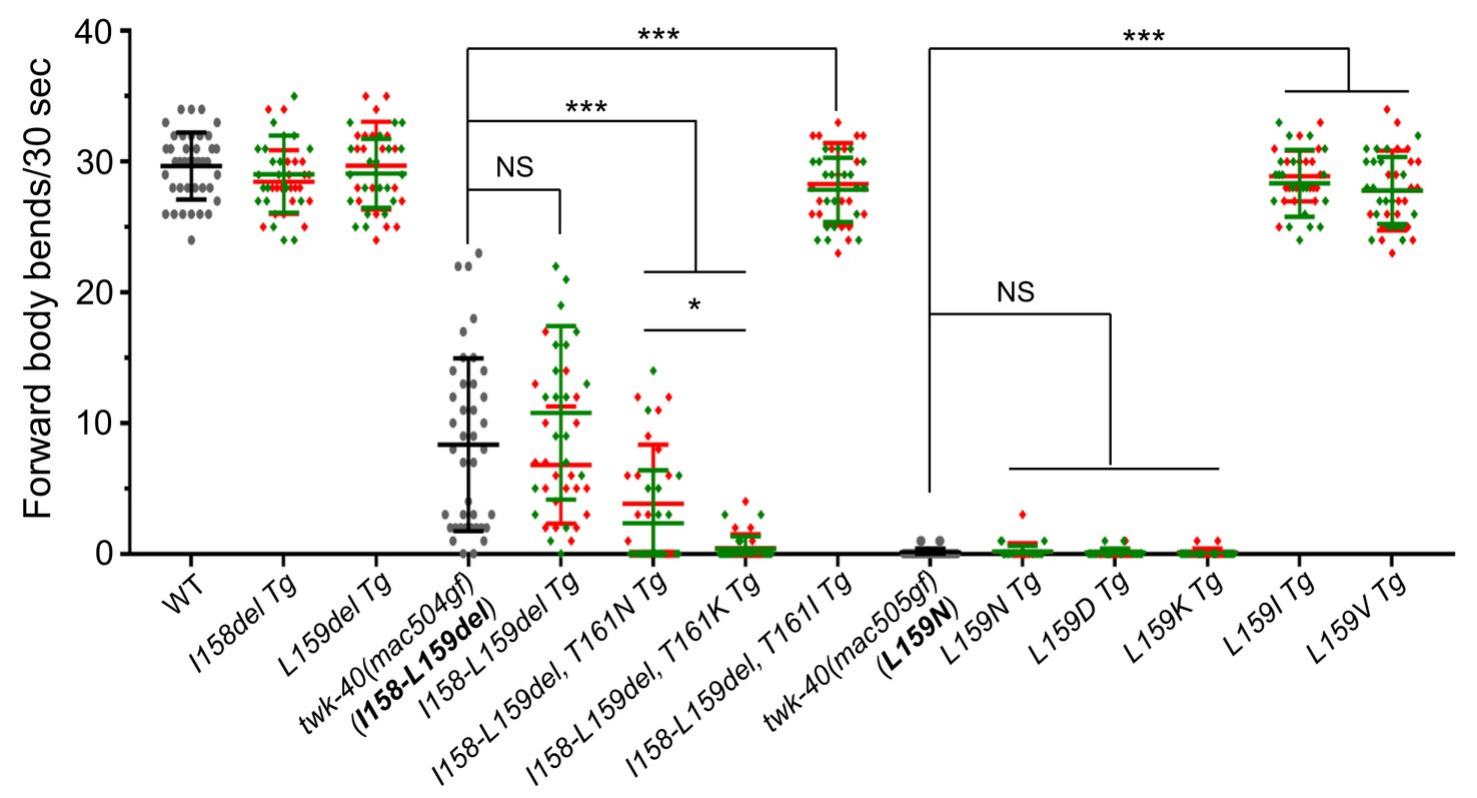
Effects of amino acid deletions and/or substitutions on TWK-40a activity. The full-length *Ptwk-40F* promoter was used to drive the expression of *twk-40a* cDNA transgenes (Tg) carrying the indicated mutations in wildtype animals. 20 young adult animals from each of the two lines were analyzed for tail touch-triggered forward body bends in 30 seconds. Data of the two lines were shown in red and green and were used together for comparison. Statistics: Bonferroni multiple comparison with one-way ANOVA. *, *p* < 0.05; ***, *p* < 0.001. NS: not significant.

We found that a *twk-40a* cDNA (I158del) transgene driven by *Ptwk-40F* failed to affect the forward locomotion of wildtype animals, so did the transgene with an L159 deletion (Fig 5).

Realizing that an L159N substitution in wildtype TWK-40a caused hyperactivity (Figs 4 and 5) and T161 in wildtype TWK-40a became T159 in TWK-40a(mac504gf) (I158-L159del) (S10A Fig), we examined whether the hyperactivity of TWK-40a(mac504gf) was related to T161 of the wildtype TWK-40a. In a *twk-40a(I158-L159del)* cDNA transgene, we mutated T161 to asparagine (N) or lysine (K). Both mutations caused paralysis in the transgenic animals (Fig 5). Interestingly, a T161I mutation in the transgene failed to do so (Fig 5).

We next examined whether substituting L159 with amino acids of different chemical properties might affect wildtype TWK-40a channel activity. Indeed, both L159D and L159K substitutions in TWK-40a caused strong paralysis (Fig 5). However, L159I or L159V substitution failed to do so (Fig 5). Together, these results suggest that the hydrophobicity/hydrophilicity of TWK-40a residue 159 may affect channel activity.

Sub-physiological extracellular K^+^ concentration is predicted to cause neuronal hyperpolarization by inducing K^+^ efflux, which might lead to defective locomotion. We tested this possibility in *C*. *elegans*. As expected, wildtype animals exhibited reduced locomotion on NGM plates in which K^+^ was substituted with Na^+^ (S12 Fig). We next explored how *twk-40* might affect this behavior.

In K^+^-deficient plates, *twk-40(lf)* mutants also exhibited reduced locomotion (S12 Fig). Surprisingly, *twk-40(mac504gf)* mutants showed weakly improved locomotion, both forward and backward, in K^+^-deficient plates (S12 Fig). Considering that human K2P TWIK-1 can conduct inward Na^+^ leak at sub-physiological extracellular K^+^ concentrations or acidic pH [61–63], further analyses of TWK-40(WT) and TWK-40(mac504gf) at low extracellular K^+^ concentration might provide new insights into TWK-40 channel activity.

## Discussion

In this study, we found that loss of function in the *K2P* gene *twk-40* suppressed the reduced body curvatures of *C*. *elegans NALCN* loss-of-function mutants. Neuron-specific *twk-40* expression and *twk-40* transcript isoforms might differentially affect the curvature and backward locomotion. We provide evidence that five other *twk* genes, including *twk-2*, *twk-17*, *twk-30*, *twk-48*, and *unc-58*, may also modulate *C*. *elegans* behavior. These *twk* genes, together with the previously described *twk-7*, exhibited variable genetic interactions with *twk-40*. Specifically, *twk-2* and *twk-40* might depend on each other for full activities. Like *twk-40*, *twk-2*, *twk-7*, and *unc-58* appeared to affect specific behaviors in opposition to *NALCN*. Finally, we detected a correlation between the hydrophobicity/hydrophilicity of TWK-40a residue 159 and the channel activity.

### K2Ps and NALCN can have opposite effects on animal behavior

K2P channels are key regulators of resting membrane potentials by conducting “leak” outward K^+^ current. The activities of K2Ps are affected by a variety of physiological stimuli [2]. NALCN has an opposite effect on resting membrane potentials by conducting primarily inward monovalent cations [6,8], and NALCN activity can be affected by different GPCRs [8,28,29]. Previous studies suggested the existence of a “leak” K^+^ channel as a balancing force for NALCN in regulating the spontaneous firing rate of mouse SNr neurons [9] or the rhythmicity of *Drosophila* clock neurons [13]. Alternatively, NALCN could regulate the excitability of pH-sensitive neurons of mouse retrotrapezoid nucleus in opposition to the background “leak” K^+^ channels [64]. These findings support the hypothesis that NALCN and certain “leak” K^+^ channels might have counterbalancing effects on resting membrane potentials and raise the question whether the “leak” K^+^ channels might be K2Ps.

Recently, Kasap et al. [33] found that pharmacological inhibition of different K^+^ channels, including K2Ps, can significantly improve the locomotion of *NALCN(lf) C*. *elegans* mutants, providing an early piece of evidence that NALCN and K2Ps might act as opposite regulators of animal behaviors. Our results are consistent with their findings. The opposite effect was observed between *twk-40(lf)* and *NALCN(lf)* and was corroborated by the suppression of the coiler phenotype of *nca-1(gf)* mutants by *twk-40(gf)*. Opposite effects on specific behaviors were observed between *NALCN* and *twk-2*, *twk-7*, or *unc-58* as well. Besides K2Ps, other channels may also affect the behaviors regulated by NALCN, *e*.*g*., increasing Ca^2+^ channel activity pharmacologically improved the locomotion of *NALCN(lf)* mutants [33]. However, the molecular mechanisms underlying the behavioral effects of these channels are largely unclear and our findings should not be interpreted that TWK-40 or other TWK channels can affect neuronal activities in opposition to NALCN. To address these questions, we need to identify the sites of action of these channels and analyze their effects on neuronal activities by multidisciplinary approaches.

### *twk-40* activities are associated with its neuron-specific expression

It is intriguing that the *Ptwk-40L* and *Ptwk-40R* promoters, which appeared to be active in different neurons, exhibited similar rescuing activities for *twk-40(lf)* suppression of *unc-80(lf)* curvature. They also showed rescuing activities for the deep curvature of *twk-40(lf)* single mutants, though *Ptwk-40L* appeared to be stronger. However, *Ptwk-40L* and *Ptwk-40R* exhibited quite different rescuing activities for the extended backward locomotion of *twk-40(lf)* mutants: *Ptwk-40R* was effective while *Ptwk-40L* was not. We recently made a similar observation about the differential effects of promoters on behaviors in our study of *C*. *elegans* avoidance response to the odorant methyl salicylate [38]. In this study, we found that a portion (*P*_*S*_*unc-79*) of the full-length *unc-79* promoter (*P*_*L*_*unc-79*) exhibited a rescuing activity like *P*_*L*_*unc-79* for the avoidance defect of *unc-79(lf)* mutants. However, *P*_*S*_*unc-79* failed to rescue the locomotion defect of *unc-79(lf)* mutants, which could be strongly rescued by *P*_*L*_*unc-79*.

Another intriguing finding was that the *Pnmr-1* promoter showed rescuing activities for both the curvature and backward locomotion phenotypes of *twk-40(lf)* mutants. However, *Pnmr-1* appeared to be active in a set of neurons that only partially overlapped with those for *Ptwk-40L*, *e*.*g*., in AVA/AVE/AVG, or with *Ptwk-40R*, *e*.*g*., in PVC.

To elucidate the underlying mechanism of these phenomena is beyond the scope of our current study. Nevertheless, it is tempting to speculate a scenario that might accommodate these findings. In this scenario, *NALCN* might affect an array of behaviors by regulating the activity of a neural network. A component of the network may affect a specific behavior redundantly with other components, or only affect component-specific behaviors, or both. TWK channels, through restricted expression and regulated activities, might be involved in compartmentalizing the expression of some behaviors oppositely regulated by NALCN.

We primarily used genetic and behavioral experiments to analyze the effects of *NALCN* and *twk* genes. We found that several tail neurons in which *twk-40* promoters were active, including PVC, DVA, DVB and DVC, did not obviously affect the curvature or backward locomotion. However, our analyses were insufficient to reveal whether one or more of the head neurons in which TWK-40 and NALCN were co-expressed, *e*.*g*., AVA, AVB and AVE, can affect these behaviors or were the sites where TWK-40 and NALCN exert opposite behavioral effects. Future experiments that can directly measure the activities of these neurons, *e*.*g*., calcium imaging or electrophysiology experiments, will provide insights into how TWK-40 and NALCN affect neuronal activities. Also, optogenetic approaches will be important for associating the behaviors affected by these channels with the neurons expressing them.

### *twk-40* transcript isoforms exhibit differential effects on the behavior

That *Ptwk-40L* (upstream of the *twk-40b* isoform) exhibited a stronger rescuing effect on the curvature phenotype of *twk-40(lf)* mutants while *Ptwk-40R* (upstream of the *twk-40c* and *twk-40a* isoforms) exhibited a stronger rescuing effect on the backward locomotion phenotype implies that *twk-40* isoforms might have functional differences. We examined this possibility using presumptive isoform-specific *twk-40* mutants.

For the curvature phenotype, *twk-40(mac564)* (*twk-40a*-specific) or *twk-40(mac557)* (*twk-40c*-specific) had no obvious effect. However, *twk-40(mac554)* (*twk-40b*-specific) caused a moderately deep curvature, and with the disruption of more isoforms, *e*.*g*., in *twk-40(mac558)* (disruption of *twk-40b* and *twk-40c*) and *twk-40(mac560)* (disruption of all three isoforms), the mutants exhibited gradually increased deep curvature. Based on these analyses, we speculate that the curvature is more significantly affected by *twk-40b* and less by *twk-40a* or *twk-40c*.

For the backward locomotion phenotype, *twk-40(mac554)* (*twk-40b*-specific), *twk-40(mac557)* (*twk-40c*-specific) or *twk-40(mac558)* (disruption of *twk-40b* and *twk-40c*) had no obvious effect. However, *twk-40(mac564)* (*twk-40a*-specific) caused a moderately extended backward locomotion, and *twk-40(mac560)* (disruption of all three isoforms) caused a more severe phenotype. Therefore, the backward locomotion appeared to be more significantly affected by *twk-40a* and less by *twk-40b* or *twk-40c*. Together, the isoform-specific mutant phenotypes are consistent with the transgene rescue results, suggesting differential behavioral effects of *twk-40* isoforms.

Caution needs to be exercised in interpreting the results of the transgenes and isoform-specific mutants. For example, transgenes may cause non-specific expression in neurons, the promoters may not be complete, and the fluorescent reporters probably did not provide a full picture about *twk-40* expression. In addition, the expression patterns of *twk-40* isoforms should not be extracted simply from the reporters, as the promoters may regulate expression of adjacent isoforms as well as distant ones. It is also possible that the presumptive *twk-40* isoform-specific mutations may affect the expression of other isoforms, as they were within the full-length promoter region and/or might affect all isoforms. Therefore, new reagents and methods that provide higher-resolution and more accurate pictures about *twk-40* expression, *e*.*g*., TWK-40 isoform-specific antibodies, isoform-specific knockin of reporters or single-copy transgene experiments, are warranted for validating our findings.

### *twk* genes may fine tune the motor behavior of *C*. *elegans*

*C*. *elegans* genome contains 47 K2P channel encoding genes [4] (www.wormbase.org). The functions of most TWK channels are largely unclear, with the exception of TWK-18 [65], SUP-9 (TWK-38) [66], TWK-7 [56,57], EGL-23 [59] and UNC-58 [58,59]. TWK-18 can be activated by high temperature [65]. SUP-9 was regulated by the transmembrane proteins UNC-93, SUP-10 and the iodotyrosine deiodinase SUP-18 [66–68]. TWK-7 affected several aspects of adaptive locomotion behavior downstream of the GαS-KIN-1/PKA pathway [56,57]. Gain-of-function mutations in EGL-23 caused egg-laying defects [59,69], while loss-of-function mutations in EGL-23 probably did not cause obviously abnormal behaviors [59]. Gain-of-function mutations in UNC-58 caused a dumpy and spasm phenotype due to hyper-activation of the body wall muscles [58,59]. However, loss-of-function phenotype of *unc-58* was unclear.

Prompted by the findings on *twk-40* in the early phase of this study, we surveyed the functions of 13 *twk* genes expressed in neurons of the motor circuit [55] (www.cengen.org) by generating loss-of-function mutations in them. Focusing on the body curvature and touch-triggered forward or backward locomotion, we found that five such *twk* genes, including *twk-2*, *twk-17*, *twk-30*, *twk-48*, and *unc-58*, might be modulators of motor behavior. Our study also confirmed the hyperactive forward locomotion of *twk-7(lf)* mutants as previously described [56,57].

The behavioral patterns exhibited by these *twk(lf)* mutants are often displayed by wildtype animals under different circumstances [70]. Therefore, we may speculate that activation or inhibition of these TWK channels might provide dynamic means for modulating *C*. *elegans* motor behavior. However, our analyses were mostly descriptive and qualitative. Extensive future studies are needed for understanding the molecular mechanisms underlying the effects of these *twk* genes.

### An interdependence between *twk-40* and *twk-2* in modulating the locomotion

We found that loss-of-function mutations in *twk-2* or *twk-40* can partially suppress the paralysis phenotype caused by gain-of-function mutations in the other. However, *twk-40* did not interact with other *twk* genes in such a manner. During the study, we also performed a screen for mutations that can suppress the paralysis phenotype of *twk-40(mac505gf)* mutants (Materials and Methods). From the screen, we isolated a potential loss of function mutation in *twk-2* predicted to change TWK-2 G181 to glutamate (E), and G181 is a conserved residue within selectivity filter 1 of K2P channels [60]. Realizing that *Ptwk-40F* and *Ptwk-2* promoters were both active in AVA, AVE, and other neurons, we postulate that *twk-40* and *twk-2* might function in same neurons for their full activities.

### Gain-of-function mutations in *twk* genes provide new insights into channel activities

Recently, Ben Soussia et al. found that changing TM2.6 to asparagine can generate hyperactivity in multiple K2Ps [60]. We generated similar mutations in TWK-40, TWK-2, TWK-7, and UNC-58, and found that they caused strong paralysis. The paralysis phenotype of *unc-58(gf)* mutants confirmed that of *unc-58(gf)* mutants generated by Ben Soussia et al. [60].

We also isolated an in-frame mutation (*mac504gf*) that deleted I158 (TM2.5) and L159 (TM2.6) of TWK-40a. *mac504gf* caused a moderately hyperactive channel. Amino acid substitutions in wildtype TWK-40a and TWK-40(mac504gf) channels and transgene phenocopy analyses suggest that the residue at TM2.6 can significantly affect channel activity. Specifically, hydrophilic residues appeared to be related to hyperactivity while hydrophobic residues appeared to be related to inactivity or highly regulated activity. Our results are consistent with recent findings that such a hydrophobic barrier on the mammalian K2P TWIK1 can oppose efficient K^+^ passage through the channel pore [71,72], and substituting the hydrophobic residue at TM2.6 of K2P channels with different amino acids can modulate the channel activities [60]. In future, detailed structural and electrophysiological analyses are warranted for understanding how these amino acid affect TWK-40 channel activity.

### A subset of *twk* genes might be recently evolved to modulate the motor behavior

**Fig 6:**
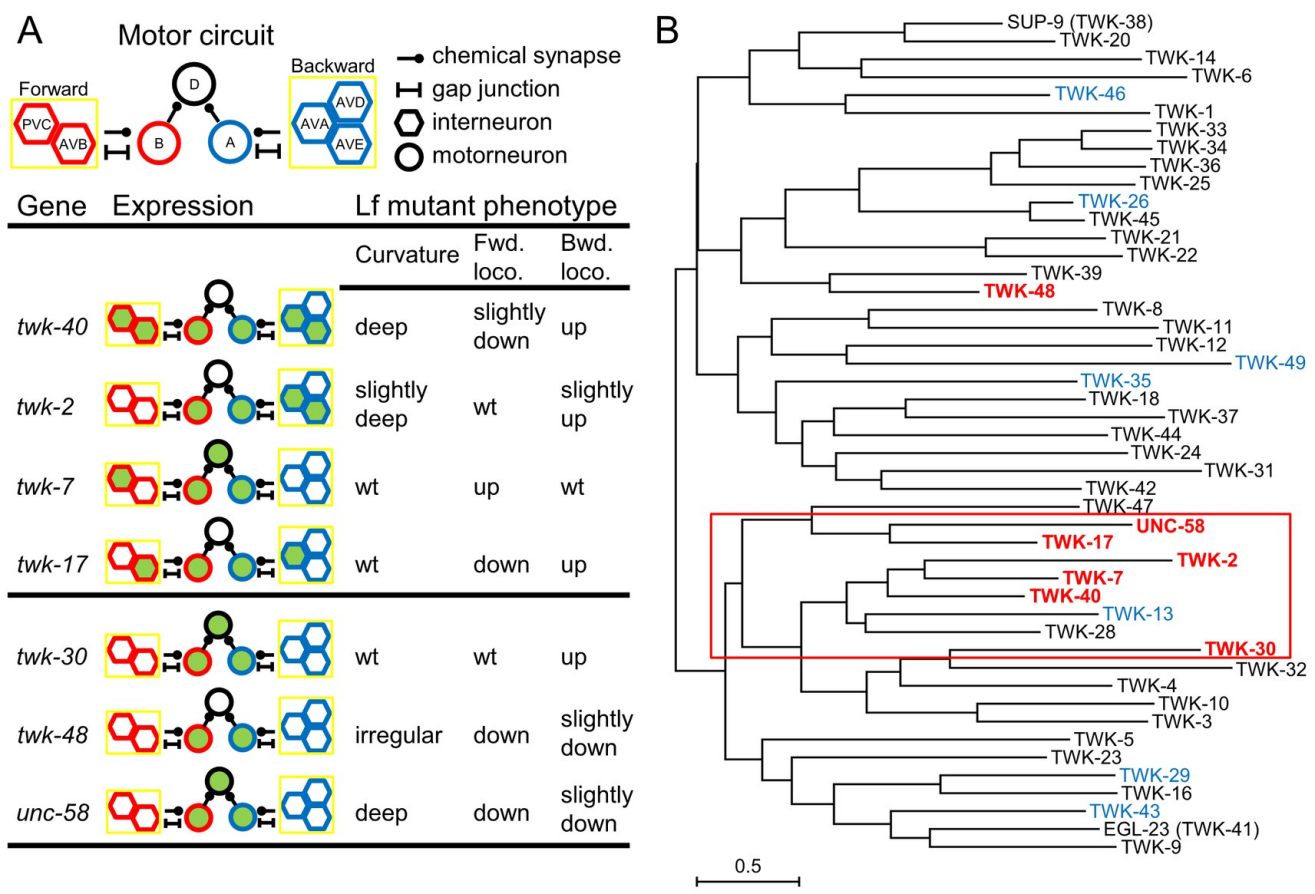
A recently diverged group of *twk* genes might modulate the motor behavior. (A) Expression pattern of *twk* genes in the motor circuit and observed behavioral phenotypes associated with loss-of-function mutations in these genes. Fwd. loco.: forward locomotion. Bwd. loco.: backward locomotion. Lf: loss-of-function. (B) A phylogenetic tree of 47 *C*. *elegans* TWK channels is drawn to scale, with branch lengths measured in the number of substitutions per site. TWKs in red exhibited obvious effects on the motor behavior. TWKs in blue were expressed in the motor circuit but obvious behavioral changes of loss-of-function mutants were not observed.

We illustrated the expression patterns of the *twk* genes with obvious behavioral effects in the motor circuit (Fig 6A). It appeared that there was a correlation between *twk*-expressing neurons and the phenotypes of the *twk(lf)* mutants. For example, among the *twk* genes with expression in interneurons (Fig 6A, *twk-40*, *twk-2*, *twk-7*, and *twk-17*), extended backward locomotion was observed if the expression was in AVA neurons (*twk-40*, *twk-2*, and *twk-17*), while reduced forward locomotion was observed if the expression was in AVB neurons (*twk-40* and *twk-17*). Alternatively, deep curvature was observed for *twk* genes expressed in AVA/AVE neurons (*twk-40* and *twk-2*). However, *twk* expression in interneurons might not be required for the mutants to show these phenotypes: loss-of-function mutations in *twk-30*, *twk-48*, and *unc-58*, genes not obviously expressed in these interneurons (based on CeNGEN), resulted in similar phenotypes (Fig 6A).

To understand the evolutionary relationship of *twk* genes, we built a phylogenetic tree of 47 *C*. *elegans* TWK proteins (Fig 6B). We found that TWK-40, TWK-2, TWK-7, TWK-17, TWK-30, and UNC-58 were all evolved from the same major branch of the tree (Fig 6B, red box). Among them, TWK-2, TWK-7, and TWK-40 were derived from two most recent evolutionary divergence. Though evolutionary convergence could also lead to such a phylogenetic pattern, it is tempting to speculate that these *twk* genes might have been generated from recent gene duplication events.

### Mammalian homologs of TWK-40 are associated with diseases

TWK-40 is homologous to human KCNK16 and KCNK-9. KCNK16 was found to be exclusively expressed in human pancreas [39]. A nucleotide polymorphism in *KCNK16* was associated with type 2 diabetes [73]. KCNK16 can negatively modulate human and mouse β-cell excitability and second-phase insulin secretion, probably through altering ER Ca^2+^ homeostasis and stress [74,75]. In retrospect, NALCN was shown to be required for M3 muscarinic receptor-activated inward current in a mouse MIN6 β-cell line [26]. These findings and our results together imply that NALCN and KCNK16 might counterbalance the excitability and insulin secretion of pancreatic β cells.

KCNK9 is highly expressed in the human brain [76,77]. NALCN is also broadly expressed in the nervous system [7,9,13,64] and can affect various neuronal activities [78]. Mutations in *NALCN* or *UNC80* are related to a group of diseases named “NALCN channelopathies” [36], which are characterized by infantile hypotonia, psychomotor retardation, characteristic facies, congenital contractures of the limbs and face, and developmental delays. A loss-of-function mutation in the imprinted *KCNK9* locus was found to be related to the maternally inherited Birk-Barel syndrome [79,80], which is characterized by congenital hypotonia, variable cleft palate, delayed development, and feeding problems. The similarity between the two types of diseases implies a convergent pathogenetic mechanism involving *KCNK9* and *NALCN*.

## Conclusions

In short, we identified *twk-40* as a novel modulator of *C*. *elegans* motor behavior and suggest that the behavioral effects of *twk-40* might be associated with its neuron-specific expression and functional differentiation of transcript isoforms. Two recent studies further analyzed TWK-40 functions in regulating the resting membrane potentials and activities of AVA and DVB neurons [81,82]. We also uncovered potential behavioral effects of five other *K2P* genes and provide genetic evidence that some K2Ps and NALCN can oppositely affect different aspects of *C*. *elegans* motor behavior. Understanding the relationship between K2Ps and NALCN might provide novel insights into the pathogenesis of a spectrum of diseases caused by mutations in NALCN and K2Ps.

## Materials and methods

### Screening for *unc-80(lf)* suppressors and mapping of mutations

Synchronized *unc-80(lf)* L4 animals were mutagenized with EMS (ethyl methanesulfonate) [58]. 400 P_0_ animals were picked to 20 NGM plates, with 20 animals per plate. We estimated that each P_0_ animal would lay at least 100 eggs within 24 hrs of becoming young adult. After these first-day F_1_ eggs (~40,000 in total) became young adults, they were bleached to generate synchronized F_2_ progeny. Once growing into young adults, F_2_ animals were examined under a dissecting microscope to identify individuals with apparently improved locomotion or body curvature. From the screen, we obtained 5 independent isolates.

All five suppressors were recessive. *mac420* and *mac422* were assigned to Chr. X because F_1_ male progeny from the cross between *unc-80(lf)* males and *unc-80(lf); mac420* or *unc-80(lf); mac422* hermaphrodites were suppressed for the defective locomotion, while F_1_ hermaphrodite progeny were not. F_1_ males from the cross between *unc-80(lf)* males and *unc-80(lf); mac424/mac425/mac426* hermaphrodites were not suppressed for their respective phenotype, suggesting that *mac424*, *mac425* and *mac426* were autosomal. Complementation test suggested that *mac420* and *mac422* might affect different genes. We did not further map *mac420* and *mac422* using SNPs.

*mac425* and *mac426* similarly suppressed the reduced body curvature of *unc-80(lf)* mutants and did not complement each other, suggesting that they might affect a same gene. We mapped *mac425* using published single nucleotide polymorphisms (SNP) [83].

To generate mapping lines, we crossed Hawaiian (CB4856) males with *unc-80(lf); mac425* hermaphrodites to generate F_1_ cross progeny (normal curvature), and their F_2_ progeny with reduced curvature, which were probably of *unc-80(lf); mac425/+* or *unc-80(lf); +/+* genotype, were picked to individual plates. Individual F_3_ progeny with suppressed curvature from different F_2_ plates were established as mapping lines.

In 10 mapping lines for *mac425*, homozygous CB4856 genotypes were not detected at *W06F12* (Chr. III: 21 cM) or *Y17D7B* (Chr. V: 18 cM. Note: *unc-80* is at Chr. V: 26 cM) but were detected at other selected SNPs in at least one line. At the same time, genomic sequencing of unoutcrossed *mac425* and *mac426* mutants found that *twk-40* was the only gene on Chr. III affected in both strains. Hence we pursued *twk-40* as the candidate gene for *mac425* and *mac426*.

The mapping of *mac424* is ongoing.

### Screening for *twk-40(mac505gf)* suppressors

We mutagenized *twk-40(mac505gf)* P_0_ animals at the L4 larval stage with EMS. From synchronized F_2_ adult progeny of ~40,000 F_1_ progeny, we isolated four recessive mutants with improved locomotion. Three isolates exhibited locomotion and body curvature like *twk-40(lf)* mutants and failed to complement *twk-40(lf)*. We postulated that these isolates probably carried intragenic loss-of-function mutations in *twk-40* and did not further analyze them.

In earlier study, we found that *twk-2((lf)* could suppress the locomotion of *twk-40(mac505gf)* mutants (Table 1). We crossed males of the fourth isolate with *twk-2(lf); twk-40(mac505gf)* hermaphrodites and found that the male progeny were still suppressed for the locomotion phenotype. Sanger sequencing identified a mutation in *twk-2* (named *mac537*). *mac537* caused a G181E change in TWK-2a. TWK-2a G181 is a conserved residue in selectivity filter 1 of K2P channels [60].

### Whole-genome sequencing

Each of the original isolates, CSM740 (*unc-80(lf); mac420*), CSM742 (*unc-80(lf); mac422*), CSM757 (*unc-80(lf); mac424*), CSM758 (*unc-80(lf); mac425*) and CSM759 (*unc-80(lf); mac426*) before outcrossing, was grown on a single 9 cm NGM plate and washed from the plates before food was completely consumed. Animals were washed twice with M9, resuspended in M9 and starved for several hours. Genomic DNAs were extracted by proteinase K digestion, followed by RNase A treatment and two rounds of phenol–chloroform extraction. Three genomic DNA libraries (380-bp inserts) were constructed by Berry Genomics Co., Ltd (Beijing) using Illumina’s paired-end protocol and paired-end sequencing (100-bp reads) was performed on the Illumina HiSeq 2000. Over 4G clean bases were mapped to the N2 genome (Wormbase release 220) after removal of duplicated reads. SNP calling was performed using Genome Analysis Toolkit (GATK) with the N2 genome as reference. 4449 (*mac420*), 4623 (*mac422*), 4633 (*mac424*), 4712 (*mac425*), and 4675 (*mac426*) sequence variants were detected in these mutants. Sequence variants shared among strains were excluded as they were likely derived from common ancestors. To enhance the stringency for mutation identification, we set the depth of reference base (WT) to be < 6 in these mutants. Exon or splice site variants carrying effective mutations with a variant quality greater than 30 were selected. Based on these criteria, we obtained 32 (*mac420*), 45 (*mac422*), 46 (*mac424*), 62 (*mac425*), and 74 (*mac426*) variants overall. The potential target genes for each mutant were further narrowed down to the mapped chromosomes.

*mac425* and *mac426* were mapped to Chr. III and might affect a same gene. Comparing sequence variants on Chr. III identified *twk-40* as the only gene that was affected in both mutants.

### Quantification of locomotion (body bends) and body curvature

Synchronized L4 animals were transferred to a new NGM plate with thin bacterium lawn and allowed to grow for 12 to 18 hours. A body bend is defined as a forward head turn or backward tail turn. Forward body bends were measured for 30 or 60 seconds after the tail of an animal was gently touched with a worm pick to trigger locomotion. Backward body bends were measured after the head of an animal was gently touched to trigger locomotion until the animal stopped or resumed forward locomotion.

Body curvature was measured based on a previously described method [30] with modification. The curvature index is the ratio of the depth (amplitude) to the period of the body waveform (Fig 1A). Young adults 18–24 hours after the mid L4 larval stage were quantified.

### Transgene experiments

Germline transgene experiments were performed as described [84].

For *twk-40* rescue experiments, the transgenic mixtures contained 20 ng/μl pPD95_86 (*Pmyo-3*::*GFP*) plasmid as co-injection marker and 20 ng/μl of the transgene of interest.

For transcriptional reporters, a transgene solution containing 20~50 ng/μl of the reporter plasmid was injected to wildtype animals.

For neuron-specific rescue experiments, the transgenic mixtures contained 10~30ng/μl *Promoter*:: *twk-40_cDNA* and 20 ng/μl pPD95_86 (*Pmyo-3*::*GFP*) plasmid or 2.5 ng/μl pCFJ90 (*Pmyo-2*::*mCherry*) plasmid [85] as co-injection marker.

We used a previously described method [86] with modifications to generate neuron-specific knockouts of *twk-40*. The transgenic mixtures contained 25 ng/μl *Promoter*::*Cas9*::*NLS*::*3’UTR*, 25 ng/μl *PU6*::*twk-40_sgRNA #1* and *#2*, and 20 ng/μl pPD95_86 (*Pmyo-3*::*GFP*) plasmid as co-injection marker.

For phenocopy experiments, transgenic mixtures contained 10~20 ng/μl *Promoter*::*twk-40a(mutations)_cDNA* and 20 ng/μl pPD95_86 (*Pmyo-3*::*GFP*) plasmid as co-injection marker.

### Generation of *twk* mutations using the CRISPR/Cas9 method

We followed the method [87] with modifications. Plasmids for microinjection were purified using Omega’s Midi Plasmid Purification kit (Omega Bio-tek). For *twk-40* insertion/deletion mutation, the injected DNA mixture contained 50 ng/μl pPD162 (*Peft-3*::*Cas9-SV40_NLS*), 25 ng/μl *PU6*::*sgRNAs* (two specific *sgRNAs* for a *twk* gene), and 20 ng/μl pPD95_86 (*Pmyo-3*::*GFP*) plasmid as co-injection marker. To generate *twk-40* knockin mutations, we included a synthesized oligo (500 nM) containing the designed mutation as repair template in the injection mixture.

We normally injected 20 to 40 wildtype P_0_ animals for knockout or knockin mutations. For *twk-40(mac469)* and *twk-40(mac472)*, we injected *unc-80(lf)* P_0_ animals. ~40 F_1_ animals with strong GFP signals in body-wall muscles (co-injection marker) were picked to individual plates, and F_2_ progeny were sequenced until the expected mutations were detected.

Two approaches were taken to generate mutations in other 14 *twk* genes. For each of *twk-2*, *twk-3*, *twk-7*, *twk-13*, *twk-17*, *twk-43*, *twk-46* and *unc-58*, knockout or knockin mutations were generated using an injection solution containing the aforementioned injection mix together with a repair template. We obtained two or more deletion/insertion mutations for each of the eight genes, one knockin mutation for *twk-2*, *twk-7* or *twk-43*, three knockin mutations for *unc-58*, and no knockin mutation for *twk-3*, *twk-13*, *twk-17* and *twk-46*. For *twk-26*, *twk-29*, *twk-30*, *twk-35*, *twk-48* and *twk-49*, we generated deletion/insertion mutations using an injection solution without repair template (S9 Table). Separate injection and examination were performed for each gene.

Target sequences of *sgRNAs* are shown in S9 Table and the sequences of repair templates for *twk-40*, *twk-2*, *twk-7*, *unc-58* and *twk-43* are shown in S10 Table. The numbers of lines examined by sequencing and the numbers of knockout lines or knockin lines obtained are shown in S9 Table.

### Confocal microscopy and identification of *twk-40*-expressing cells

Confocal pictures of fluorescent transgenic animals were taken with a Zeiss 880 confocal microscope (10X and 63X objective). Multiple animals from each of two transgenic lines were observed for consistency of reporter expression and a subset of animals with strong fluorescence were examined further for neuronal identification. Pictures shown were representative images. We determined the identities of *twk-40*-expressing cells labeled with *Ptwk-40*::*reporters* by examining the anatomical positions, morphologies, patterns and neighboring cells and comparing to the descriptions at www.wormatlas.org. The identities of some neurons were further verified by co-labeling experiments with reporters driven by previously described promoters.

### Potassium-deficiency assay

NGM plates were prepared by replacing K_2_HPO_4_/ KH_2_PO_4_ with equal molar amount of Na_2_HPO_4_/ NaH_2_PO_4_. Synchronized L1 animals were allowed to grow on the K^+^-deficient plates until reaching the L4 larval stage. The animals were then transferred to fresh K^+^-deficient NGM plates with thin bacterium lawn. Touch-triggered body bends were measured in 18 to 24 hours.

### Neuronal expression pattern based on CeNGEN

We followed the instruction at www.cengen.org to search for the graphed expression of each *twk* gene in *C*. *elegans* neurons. The top 20 neuronal classes in which a *twk* gene was expressed were listed in S4 Table.

### Phylogenetic tree

Maximum likelihood phylogenetic tree was constructed with the Molecular Evolutionary Genetics Analysis software MEGA X [88].

### Statistics

*P* values were determined by two-tailed unpaired Student’s *t*-test for pairwise comparison or Bonferroni multiple comparison with one-way ANOVA for multiple comparison using GraphPad Prism 7.0 software.

Strains used in this study and plasmid constructions are shown in S1 Text. Raw data are shown in S1 Data.

## Supporting information

S1 Fig Behavioral quantification of *unc-80(lf)* suppressors and *nca(lf)* mutants. (A) Touch-triggered forward locomotion of *unc-80(lf); sup* mutants. *mac420*, *mac422* and *mac424* could significantly improve the defective locomotion of *unc-80(lf)* mutants. (B) Curvature indices of *nca-1(lf)* single, *nca-2(lf)* single and *nca-2(lf); nca-1(lf)* double mutants. 40 animals were quantified for each genotype. Statistics: Bonferroni multiple comparison with one-way ANOVA. ***, *p* < 0.001.
(TIF)

S2 Fig Protein sequence alignment of TWK-40 and homologs. The four transmembrane domains are enclosed in blue boxes, and the two pore domains are in red boxes. The amino acid at TM2.6 is indicated with a red arrowhead. The first 50 amino acids of the *Drosophila* CG43155 were omitted. C-terminal regions of TWK-40a (aa 338–393), *Drosophila* CG43155 (aa 364–411), *M*. *musculus* KCNK3 (aa 285–409), *H*. *sapiens* KCNK9 (aa 287–401) and *H*. *sapiens* KCNK16 (aa 260–262) were not included in the alignment.
(TIF)

S3 Fig *twk-40* promoters were active in the nervous system. (A) The genomic positions of *Ptwk-40F*, *Ptwk-40L* and *Ptwk-40R* promoters. (B) GFP driven by the *Ptwk-40L* promoter labeled some head neurons, ventral nerve cord and tail neurons. Ventral cord motor neurons were not obviously labeled. (C) mCherry driven by the *Ptwk-40R* promoter labeled multiple head neurons, ventral nerve cord, ventral motor neurons and tail neurons. a: anterior; v: ventral.
(TIF)

S4 Fig *Ptwk-40R* and *Ptwk-40L* appeared to be active in mostly non-overlapping neurons. (A) Confocal pictures of head neurons labeled by *Ptwk-40R*::*mCherry* (left panel) and *Ptwk-40L*::*GFP* (middle panel). The merged picture was shown on the right. (B) Confocal pictures of the same animal as in (A) at a different focal plane. (C) A tail neuron(s) at the position of DVA/DVB/DVC neurons was co-labeled by mCherry and GFP. (D) Ventral nerve cord in the middle region of an animal showing the mCherry-labeled motor neurons and GFP-labeled cord. For all pictures, a: anterior; v: ventral.
(TIF)

S5 Fig *Ptwk-40L::mCherry* and *Punc-80::GFP* appeared to co-label AVA, AVE, AVB and RIS neurons. (A) Confocal pictures of adult head neurons expressing the *Ptwk-40L*::*mCherry* transgene (left panel) and the *Punc-80*::*GFP* transgene (middle panel). (B) Confocal picture of the same animal as in (A) on a different focal plane. (C) A tail neuron(s) at the position of DVA/DVB/DVC was co-labeled by mCherry (left panel) and GFP (middle panel). The merged pictures were shown on the right. For all pictures, a: anterior; v: ventral.
(TIF)

S6 Fig *Ptwk-40R::mCherry* and *Punc-80::GFP* appeared to co-label a subset of unidentified interneurons. (A) Confocal pictures of adult head neurons expressing the *Ptwk-40R*::*mCherry* transgene (left panel) and the *Punc-80*::*GFP* transgene (middle panel). The merged picture was shown on the right. (B) Confocal picture of the same animal as in (A) at a different focal plane. (C) A tail neuron(s) at the position of DVA/DVB/DVC appeared to be co-labeled by mCherry (left panel) and GFP (middle panel). (D) Ventral cord in the middle region of an animal showing several motor neurons co-labeled by mCherry and GFP. For all pictures, a: anterior; v: ventral.
(TIF)

S7 Fig *twk-40* promoters and the *nmr-1* promoter appeared to co-label some neurons. (A) Confocal pictures of adult head neurons expressing the *Ptwk-40R*::*GFP* transgene (left panel) and the *Pnmr-1*::*mCherry* transgene (middle panel). The merged picture was shown on the right. (B) Confocal pictures of adult tail neurons expressing the *Ptwk-40R*::*GFP* transgene (left panel) and the *Pnmr-1*::*mCherry* transgene (middle panel). (C) Confocal pictures of adult head neurons expressing the *Ptwk-40L*::*GFP* transgene (left panel) and the *Pnmr-1*::*mCherry* transgene (middle panel). For all pictures, a: anterior; v: ventral.
(TIF)

S8 Fig *twk-40* promoters were active in DVA, DVB and DVC nervous. (A) Confocal pictures of adult tail neurons expressing the *Ptwk-40F*::*mCherry* transgene (left panel) and the *Ptrp-4*::*GFP* transgene (middle panel). (B) Confocal pictures of adult tail neurons expressing the *Ptwk-40F*::*mCherry* transgene (left panel) and the *Plim-6*^*int4*^::*GFP* transgene (middle panel). (C) Confocal pictures of adult tail neurons expressing the *Ptwk-40L*::*mCherry* transgene (left panel) and the *Plim-6*^*int4*^::*GFP* transgene (middle panel). (D) Confocal pictures of adult tail neurons expressing the *Ptwk-40R*::*mCherry* transgene (left panel) and the *Plim-6*^*int4*^::*GFP* transgene (middle panel). The merged pictures were shown on the right. For all pictures, a: anterior; v: ventral.
(TIF)

S9 Fig Mutations affecting *twk-40* transcript isoforms. (A) List of presumptive *twk-40* isoform-specific mutations. (B) Genomic positions of *twk-40* isoform-specific mutations.
(TIF)

S10 Fig TWK 2^nd^ transmembrane domain sequence alignment and phenotypes of TWK gain-of-function mutants. (A) Alignment of the 2^nd^ transmembrane domains of indicated TWK channels. TM2.6 was substituted with asparagine (N). (B) Representative pictures of *twk(gf)* mutants.
(TIF)

S11 Fig The *Ptwk-40F* and *Ptwk-2* promoters appeared to be active in same neurons. (A) Confocal pictures of adult head neurons expressing the *Ptwk-40F*::*GFP* transgene (left panel) and a *Ptwk-2*::*mCherry* transgene (middle panel). (B) An unidentified tail neuron(s) at the position of DVA/DVB/DVC was co-labeled by GFP (left panel) and mCherry (middle panel). The merged pictures were shown on the right. (C) Ventral cord in the middle region of a transgenic animal showing two motor neurons co-labeled by GFP and mCherry. For all pictures, a: anterior; v: ventral.
(TIF)

S12 Fig TWK-40(mac504gf) mutants exhibited improved locomotion in K^+^-deficient environment. 40 animals were quantified for each assay condition. Statistics: two-tailed unpaired Student’s *t*-test. ***, *p* < 0.001.
(TIF)

S1 Table List of *unc-80(lf)* suppressors and deleterious genetic variations identified in *unc-80(lf); mac425/mac426* mutants. Chromosomal locations of the suppressors are shown at the top. Genetic variations identified from genomic sequencing of unoutcrossed *unc-80(lf); mac425* and *unc-80(lf); mac426* mutants are shown at the bottom.
(XLSX)

S2 Table List of *twk-40a* mutations. (XLSX)

S3 Table List of *C. elegans twk* genes and their expression in the motor circuit neurons based on the CeNGEN project (www.cengen.org). Genes in red were expressed in the motor circuit and exhibited detectable effects on the motor behavior. Genes in blue were expressed in the motor circuit and did not obviously affect the motor behavior. Genes in bold black were previously studied but not analyzed in this study. Behaviors of loss-of-function mutants were considered here. *twk-40* was not annotated to be obviously expressed in PVC neurons at CeNGEN. *twk-3* was chosen as a presumptive negative control due to its lack of obvious expression in the motor circuit. The relative expression levels of *twk* genes (based on CeNGEN) in each neuronal type are qualitatively indicated with the numbers of asterisks: *, weak; **, moderate; ***, strong.
(XLSX)

S4 Table List of mutations and neuronal expression patterns of 14 *twk* genes. We used the CRISPR/Cas9 method to generate mutations in these *twk* genes. For each gene, top 20 neuron classes with high to low transcript signals (www.cengen.org) were listed. Mutations in bold were used as reference alleles. Key neurons in the motor circuit are shown in red.
(XLSX)

S5 Table Transgene rescue of *twk(lf)* mutant phenotype. Wildtype transgenes under control of the *unc-119* promoter were used. 20 animals from each of two transgenic lines were quantified and the data were used together for comparison. Obvious phenotypes of *twk-17(lf)* and *twk-48(lf)* mutants other than the body curvature or locomotion were not quantified due to strong rescuing effects by the transgenes. Statistics: two-tailed unpaired Student’s *t*-test. ***, *p* < 0.001. NA: not applicable.
(XLSX)

S6 Table Curvature indices and backward body bends of *twk* loss-of-function mutants without obviously abnormal behavioral phenotypes. 40 animals were measured in each dataset. Statistics: Bonferroni multiple comparison with one-way ANOVA, ***, *p* < 0.001.
(XLSX)

S7 Table Genetic interactions between *twk* loss-of-function and presumptive gain-of-function mutations. (XLSX)

S8 Table List of PCR primers for generating promoters, transgenes, and the indicated mutations. (XLSX)

S9 Table *twk* target sequences used in CRISPR/Cas9-based mutagenesis. For all *twk* genes, two sgRNAs were used simultaneously. The use of *twk-40* sgRNAs was as follows: *twk-40a sgRNA* # a1 and # a2 together with a repair template (S10 Table) for *mac564*; *twk-40a sgRNA* # a3 and # a4 for *mac469*, *mac472* and neuron-specific knockouts*; twk-40a sgRNA* # a5 and # a6 together with a repair template for *mac504* and *mac505*; *twk-40b sgRNA* # b1 and # b2 for *mac554* and *mac555; twk-40c sgRNA* # c1 and # c2 for *mac556* and *mac557; twk-40b sgRNA* # b2 and *twk-40c sgRNA* # c2 *for mac558*, *mac559* and *mac560*. For each gene or *twk-40* isoform, the number of transgenic lines examined, and the number of lines carrying the indicated mutations are shown.
(XLSX)

S10 Table Repair template sequences for introducing *twk-40a* isoform-specific mutations and TWK TM2.6>N mutations. The *twk-40a* #1 repair template was used for generating *mac564* and *twk-40a* #2 for *mac505*. Nucleotides in red are for introducing the missense mutation and nucleotides in blue are silent mismatch mutations of the target sequences.
(XLSX)

S1 Movie N2_Forward. (MP4)

S2 Movie *twk-40(lf)_*Forward. (MP4)

S3 Movie N2_Backward. (MP4)

S4 Movie *twk-40(lf)_*Backward. (MP4)

S5 Movie *twk-2(lf)_*Forward. (MP4)

S6 Movie *twk-17(lf)_*Forward. (MP4)

S7 Movie *twk-30(lf)_*Backward. (MP4)

S8 Movie *twk-48(lf)_*Forward. (MP4)

S9 Movie *unc-58(lf)_*Forward. (MP4)

S10 Movie *twk-2(lf); twk-40(lf)_*Forward. (MP4)

S11 Movie *twk-7(lf) twk-40(lf)_*Forward. (MP4)

S12 Movie *twk-40(lf); twk-17(lf)_*Forward. (MP4)

S13 Movie *twk-48(lf) twk-40(lf)_*Forward. (MP4)

S14 Movie *twk-48(lf) twk-40(lf)_*Backward. (MP4)

S15 Movie *twk-40(lf); unc-58(lf)_*Forward. (MP4)

S16 Movie *twk-40(lf); unc-58(lf)_*Backward. (MP4)

S17 Movie *twk-2(lf); twk-40(gf)_*Forward. (MP4)

S18 Movie *twk-2(lf); twk-40(gf)_*Backward. (MP4)

S19 Movie *twk-2(gf); twk-40(lf)_*Forward. (MP4)

S20 Movie *twk-2(gf); twk-40(lf)_*Backward. (MP4)

S1 Text Strains and plasmid constructions. (DOCX)

S1 Data Raw data for curvature indices and body bends. (XLSX)

## References

[1] Hille B. Ion Channels of Excitable Membranes. 3rd edition. Sunderland, Mass: Sinauer Associates is an imprint of Oxford University Press; 2001.

[2] Feliciangeli S, Chatelain FC, Bichet D, Lesage F. The family of K2P channels: salient structural and functional properties. J Physiol. 2015;593: 2587–603. doi: 10.1113/jphysiol.2014.287268 25530075 PMC4500345

[3] Goldstein SAN, Bayliss DA, Kim D, Lesage F, Plant LD, Rajan S. International Union of Pharmacology. LV. Nomenclature and Molecular Relationships of Two-P Potassium Channels. Pharmacol Rev. 2005;57: 527–540. doi: 10.1124/pr.57.4.12 16382106

[4] Hobert O. The neuronal genome of *Caenorhabditis elegans*. WormBook. 2013; 1–106. doi: 10.1895/wormbook.1.161.1 24081909 PMC4781646

[5] Enyedi P, Czirják G. Molecular background of leak K+ currents: two-pore domain potassium channels. Physiol Rev. 2010;90: 559–605. doi: 10.1152/physrev.00029.2009 20393194

[6] Chua HC, Wulf M, Weidling C, Rasmussen LP, Pless SA. The NALCN channel complex is voltage sensitive and directly modulated by extracellular calcium. Sci Adv. 2020;6: eaaz3154. doi: 10.1126/sciadv.aaz3154 32494638 PMC7182417

[7] Lu B, Su Y, Das S, Liu J, Xia J, Ren D. The neuronal channel NALCN contributes resting sodium permeability and is required for normal respiratory rhythm. Cell. 2007;129: 371–83. doi: 10.1016/j.cell.2007.02.041 17448995

[8] Ren D. Sodium leak channels in neuronal excitability and rhythmic behaviors. Neuron. 2011;72: 899–911. doi: 10.1016/j.neuron.2011.12.007 22196327 PMC3247702

[9] Lutas A, Lahmann C, Soumillon M, Yellen G. The leak channel NALCN controls tonic firing and glycolytic sensitivity of substantia nigra pars reticulata neurons. Elife. 2016;5: e15271. doi: 10.7554/eLife.15271 27177420 PMC4902561

[10] Yeh SY, Huang WH, Wang W, Ward CS, Chao ES, Wu Z, et al. Respiratory Network Stability and Modulatory Response to Substance P Require Nalcn. Neuron. 2017;94: 294–303 e4. doi: 10.1016/j.neuron.2017.03.024 28392070 PMC5702257

[11] Funato H, Miyoshi C, Fujiyama T, Kanda T, Sato M, Wang Z, et al. Forward-genetics analysis of sleep in randomly mutagenized mice. Nature. 2016;539: 378–383. doi: 10.1038/nature20142 27806374 PMC6076225

[12] Nash HA, Scott RL, Lear BC, Allada R. An unusual cation channel mediates photic control of locomotion in *Drosophila*. Current biology: CB. 2002;12: 2152–8. doi: 10.1016/s0960-9822(02)01358-1 12498692

[13] Flourakis M, Kula-Eversole E, Hutchison AL, Han TH, Aranda K, Moose DL, et al. A Conserved Bicycle Model for Circadian Clock Control of Membrane Excitability. Cell. 2015;162: 836–48. doi: 10.1016/j.cell.2015.07.036 26276633 PMC4537776

[14] Lear BC, Lin JM, Keath JR, McGill JJ, Raman IM, Allada R. The ion channel narrow abdomen is critical for neural output of the *Drosophila* circadian pacemaker. Neuron. 2005;48: 965–76. doi: 10.1016/j.neuron.2005.10.030 16364900

[15] Moose DL, Haase SJ, Aldrich BT, Lear BC. The Narrow Abdomen Ion Channel Complex Is Highly Stable and Persists from Development into Adult Stages to Promote Behavioral Rhythmicity. Front Cell Neurosci. 2017;11: 159. doi: 10.3389/fncel.2017.00159 28634443 PMC5459923

[16] Zhang L, Chung BY, Lear BC, Kilman VL, Liu Y, Mahesh G, et al. DN1(p) circadian neurons coordinate acute light and PDF inputs to produce robust daily behavior in *Drosophila*. Curr Biol. 2010;20: 591–599. doi: 10.1016/j.cub.2010.02.056 20362452 PMC2864127

[17] Jospin M, Watanabe S, Joshi D, Young S, Hamming K, Thacker C, et al. UNC-80 and the NCA ion channels contribute to endocytosis defects in synaptojanin mutants. Current biology: CB. 2007;17: 1595–600. doi: 10.1016/j.cub.2007.08.036 17825559

[18] Yeh E, Ng S, Zhang M, Bouhours M, Wang Y, Wang M, et al. A putative cation channel, NCA-1, and a novel protein, UNC-80, transmit neuronal activity in *C*. *elegans*. PLoS biology. 2008;6: e55. doi: 10.1371/journal.pbio.0060055 18336069 PMC2265767

[19] Gao S, Xie L, Kawano T, Po MD, Guan S, Zhen M, et al. The NCA sodium leak channel is required for persistent motor circuit activity that sustains locomotion. Nature communications. 2015;6: 6323. doi: 10.1038/ncomms7323 25716181

[20] Humphrey JA, Hamming KS, Thacker CM, Scott RL, Sedensky MM, Snutch TP, et al. A putative cation channel and its novel regulator: cross-species conservation of effects on general anesthesia. Current biology: CB. 2007;17: 624–9. doi: 10.1016/j.cub.2007.02.037 17350263

[21] Pierce-Shimomura JT, Chen BL, Mun JJ, Ho R, Sarkis R, McIntire SL. Genetic analysis of crawling and swimming locomotory patterns in *C*. *elegans*. Proceedings of the National Academy of Sciences of the United States of America. 2008;105: 20982–7. doi: 10.1073/pnas.0810359105 19074276 PMC2634943

[22] Speca DJ, Chihara D, Ashique AM, Bowers MS, Pierce-Shimomura JT, Lee J, et al. Conserved role of *unc-79* in ethanol responses in lightweight mutant mice. PLoS genetics. 2010;6. doi: 10.1371/journal.pgen.1001057 20714347 PMC2920847

[23] Huang H, Hayden DJ, Zhu C-T, Bennett HL, Venkatachalam V, Skuja LL, et al. Gap Junctions and NCA Cation Channels Are Critical for Developmentally Timed Sleep and Arousal in *Caenorhabditis elegans*. Genetics. 2018;210: 1369–1381. doi: 10.1534/genetics.118.301551 30323068 PMC6283151

[24] Lu B, Su Y, Das S, Wang H, Wang Y, Liu J, et al. Peptide neurotransmitters activate a cation channel complex of NALCN and UNC-80. Nature. 2009;457: 741–4. doi: 10.1038/nature07579 19092807 PMC2810458

[25] Wang H, Ren D. UNC80 functions as a scaffold for Src kinases in NALCN channel function. Channels (Austin). 2009;3: 161–3. doi: 10.4161/chan.3.3.8853 19535918 PMC2810456

[26] Swayne LA, Mezghrani A, Varrault A, Chemin J, Bertrand G, Dalle S, et al. The NALCN ion channel is activated by M3 muscarinic receptors in a pancreatic beta-cell line. EMBO Rep. 2009;10: 873–80. doi: 10.1038/embor.2009.125 19575010 PMC2710536

[27] Lu B, Zhang Q, Wang H, Wang Y, Nakayama M, Ren D. Extracellular calcium controls background current and neuronal excitability via an UNC79-UNC80-NALCN cation channel complex. Neuron. 2010;68: 488–99. doi: 10.1016/j.neuron.2010.09.014 21040849 PMC2987630

[28] Philippart F, Khaliq ZM. Gi/o protein-coupled receptors in dopamine neurons inhibit the sodium leak channel NALCN. Elife. 2018;7. doi: 10.7554/eLife.40984 30556810 PMC6305199

[29] Topalidou I, Cooper K, Pereira L, Ailion M. Dopamine negatively modulates the NCA ion channels in *C*. *elegans*. PLoS genetics. 2017;13: e1007032. doi: 10.1371/journal.pgen.1007032 28968387 PMC5638609

[30] Topalidou I, Chen PA, Cooper K, Watanabe S, Jorgensen EM, Ailion M. The NCA-1 and NCA-2 Ion Channels Function Downstream of Gq and Rho To Regulate Locomotion in *Caenorhabditis elegans*. Genetics. 2017;206: 265–282. doi: 10.1534/genetics.116.198820 28325749 PMC5419474

[31] Hoyt JM, Wilson SK, Kasa M, Rise JS, Topalidou I, Ailion M. The SEK-1 p38 MAP Kinase Pathway Modulates Gq Signaling in *Caenorhabditis elegans*. G3 (Bethesda). 2017;7: 2979–2989. doi: 10.1534/g3.117.043273 28696924 PMC5592925

[32] Bouhours M, Po MD, Gao S, Hung W, Li H, Georgiou J, et al. A co-operative regulation of neuronal excitability by UNC-7 innexin and NCA/NALCN leak channel. Mol Brain. 2011;4: 16. doi: 10.1186/1756-6606-4-16 21489288 PMC3102621

[33] Kasap M, Bonnett K, Aamodt EJ, Dwyer DS. Akinesia and freezing caused by Na+ leak-current channel (NALCN) deficiency corrected by pharmacological inhibition of K+ channels and gap junctions. J Comp Neurol. 2017;525: 1109–1121. doi: 10.1002/cne.24119 27636205

[34] Xie L, Gao S, Alcaire SM, Aoyagi K, Wang Y, Griffin JK, et al. NLF-1 delivers a sodium leak channel to regulate neuronal excitability and modulate rhythmic locomotion. Neuron. 2013;77: 1069–82. doi: 10.1016/j.neuron.2013.01.018 23522043

[35] Lear BC, Darrah EJ, Aldrich BT, Gebre S, Scott RL, Nash HA, et al. UNC79 and UNC80, putative auxiliary subunits of the NARROW ABDOMEN ion channel, are indispensable for robust circadian locomotor rhythms in *Drosophila*. PloS one. 2013;8: e78147. doi: 10.1371/journal.pone.0078147 24223770 PMC3818319

[36] Bramswig NC, Bertoli-Avella AM, Albrecht B, Al Aqeel AI, Alhashem A, Al-Sannaa N, et al. Genetic variants in components of the NALCN-UNC80-UNC79 ion channel complex cause a broad clinical phenotype (NALCN channelopathies). Hum Genet. 2018;137: 753–768. doi: 10.1007/s00439-018-1929-5 30167850 PMC6671679

[37] Luo J, Xu Z, Tan Z, Zhang Z, Ma L. Neuropeptide Receptors NPR-1 and NPR-2 Regulate *Caenorhabditis elegans* Avoidance Response to the Plant Stress Hormone Methyl Salicylate. Genetics. 2015;199: 523–31. doi: 10.1534/genetics.114.172239 25527285 PMC4317659

[38] Zhou C, Luo J, He X, Zhou Q, He Y, Wang X, et al. The NALCN Channel Regulator UNC-80 Functions in a Subset of Interneurons To Regulate *Caenorhabditis elegans* Reversal Behavior. G3 (Bethesda). 2020;10: 199–210. doi: 10.1534/g3.119.400692 31690562 PMC6945035

[39] Girard C, Duprat F, Terrenoire C, Tinel N, Fosset M, Romey G, et al. Genomic and functional characteristics of novel human pancreatic 2P domain K(+) channels. Biochem Biophys Res Commun. 2001;282: 249–256. doi: 10.1006/bbrc.2001.4562 11263999

[40] Kim Y, Bang H, Kim D. TASK-3, a new member of the tandem pore K(+) channel family. The Journal of biological chemistry. 2000;275: 9340–7. doi: 10.1074/jbc.275.13.9340 10734076

[41] Rajan S, Wischmeyer E, Xin Liu G, Preisig-Muller R, Daut J, Karschin A, et al. TASK-3, a novel tandem pore domain acid-sensitive K+ channel. An extracellular histiding as pH sensor. The Journal of biological chemistry. 2000;275: 16650–7. doi: 10.1074/jbc.M000030200 10747866

[42] Brockie PJ, Mellem JE, Hills T, Madsen DM, Maricq AV. The *C*. *elegans* Glutamate Receptor Subunit NMR-1 Is Required for Slow NMDA-Activated Currents that Regulate Reversal Frequency during Locomotion. Neuron. 2001;31: 617–630. doi: 10.1016/s0896-6273(01)00394-4 11545720

[43] Kano T, Brockie PJ, Sassa T, Fujimoto H, Kawahara Y, Iino Y, et al. Memory in *Caenorhabditis elegans* is Mediated By NMDA-Type Ionotropic Glutamate Receptors. Curr Biol. 2008;18: 1010–1015. doi: 10.1016/j.cub.2008.05.051 18583134 PMC2645413

[44] Zhen M, Samuel ADT. *C*. *elegans* locomotion: small circuits, complex functions. Curr Opin Neurobiol. 2015;33: 117–126. doi: 10.1016/j.conb.2015.03.009 25845627

[45] Chalfie M, Sulston JE, White JG, Southgate E, Thomson JN, Brenner S. The neural circuit for touch sensitivity in *Caenorhabditis elegans*. The Journal of neuroscience: the official journal of the Society for Neuroscience. 1985;5: 956–64. 10.1523/JNEUROSCI.05-04-00956.1985 PMC6565008 3981252

[46] Wicks SR, Roehrig CJ, Rankin CH. A dynamic network simulation of the nematode tap withdrawal circuit: predictions concerning synaptic function using behavioral criteria. J Neurosci. 1996;16: 4017–4031. doi: 10.1523/JNEUROSCI.16-12-04017.1996 8656295 PMC6578605

[47] Riddle DL, Blumenthal T, Meyer BJ, Priess JR. Mechanosensory Control of Locomotion. *C*. *elegans* II. 2nd edition. Cold Spring Harbor Laboratory Press; 1997. Available: https://www.ncbi.nlm.nih.gov/books/NBK20005/21413221

[48] Ardiel EL, Rankin CH. Cross-referencing online activity with the connectome to identify a neglected but well-connected neuron. Current Biology. 2015;25: R405–R406. doi: 10.1016/j.cub.2015.03.043 25989076

[49] Li W, Feng Z, Sternberg PW, Shawn Xu XZ. A *C*. *elegans* stretch receptor neuron revealed by a mechanosensitive TRP channel homologue. Nature. 2006;440: 684–687. doi: 10.1038/nature04538 16572173 PMC2865900

[50] Walker RG, Willingham AT, Zuker CS. A *Drosophila* mechanosensory transduction channel. Science. 2000;287: 2229–2234. doi: 10.1126/science.287.5461.2229 10744543

[51] Hart MP, Hobert O. Neurexin controls plasticity of a mature, sexually dimorphic neuron. Nature. 2018;553: 165–170. doi: 10.1038/nature25192 29323291 PMC5968453

[52] Maduro M, Pilgrim D. Conservation of function and expression of *unc-119* from two *Caenorhabditis* species despite divergence of non-coding DNA. Gene. 1996;183: 77–85. doi: 10.1016/s0378-1119(96)00491-x 8996090

[53] Jospin M, Qi YB, Stawicki TM, Boulin T, Schuske KR, Horvitz HR, et al. A Neuronal Acetylcholine Receptor Regulates the Balance of Muscle Excitation and Inhibition in *Caenorhabditis elegans*. PLoS Biol. 2009;7. doi: 10.1371/journal.pbio.1000265 20027209 PMC2787625

[54] Shaham S, Bargmann CI. Control of neuronal subtype identity by the *C*. *elegans* ARID protein CFI-1. Genes Dev. 2002;16: 972–983. doi: 10.1101/gad.976002 11959845 PMC152356

[55] Hammarlund M, Hobert O, Miller DM, Sestan N. The CeNGEN Project: The Complete Gene Expression Map of an Entire Nervous System. Neuron. 2018;99: 430–433. doi: 10.1016/j.neuron.2018.07.042 30092212 PMC6576255

[56] Gottschling DC, Doring F, Luersen K. Locomotion Behavior Is Affected by the GalphaS Pathway and the Two-Pore-Domain K(+) Channel TWK-7 Interacting in GABAergic Motor Neurons in *Caenorhabditis elegans*. Genetics. 2017;206: 283–297. doi: 10.1534/genetics.116.195669 28341653 PMC5419475

[57] Luersen K, Gottschling DC, Doring F. Complex Locomotion Behavior Changes Are Induced in *Caenorhabditis elegans* by the Lack of the Regulatory Leak K+ Channel TWK-7. Genetics. 2016;204: 683–701. doi: 10.1534/genetics.116.188896 27535928 PMC5068855

[58] Brenner S. The genetics of *Caenorhabditis elegans*. Genetics. 1974;77: 71–94. doi: 10.1093/genetics/77.1.71 4366476 PMC1213120

[59] Reiner DJ, Weinshenker D, Thomas JH. Analysis of Dominant Mutations Affecting Muscle Excitation in *Caenorhabditis elegans*. Genetics. 1995;141: 961–976. doi: 10.1093/genetics/141.3.961 8582640 PMC1206858

[60] Ben Soussia I, El Mouridi S, Kang D, Leclercq-Blondel A, Khoubza L, Tardy P, et al. Mutation of a single residue promotes gating of vertebrate and invertebrate two-pore domain potassium channels. Nat Commun. 2019;10: 787. doi: 10.1038/s41467-019-08710-3 30770809 PMC6377628

[61] Chatelain FC, Bichet D, Douguet D, Feliciangeli S, Bendahhou S, Reichold M, et al. TWIK1, a unique background channel with variable ion selectivity. Proc Natl Acad Sci U S A. 2012;109: 5499–5504. doi: 10.1073/pnas.1201132109 22431633 PMC3325654

[62] Chen H, Chatelain FC, Lesage F. Altered and dynamic ion selectivity of K+ channels in cell development and excitability. Trends Pharmacol Sci. 2014;35: 461–469. doi: 10.1016/j.tips.2014.06.002 25023607 PMC4467785

[63] Ma L, Zhang X, Chen H. TWIK-1 two-pore domain potassium channels change ion selectivity and conduct inward leak sodium currents in hypokalemia. Sci Signal. 2011;4: ra37. doi: 10.1126/scisignal.2001726 21653227

[64] Shi Y, Abe C, Holloway BB, Shu S, Kumar NN, Weaver JL, et al. Nalcn Is a “Leak” Sodium Channel That Regulates Excitability of Brainstem Chemosensory Neurons and Breathing. The Journal of neuroscience: the official journal of the Society for Neuroscience. 2016;36: 8174–87. doi: 10.1523/JNEUROSCI.1096-16.2016 27488637 PMC4971364

[65] Kunkel MT, Johnstone DB, Thomas JH, Salkoff L. Mutants of a temperature-sensitive two-P domain potassium channel. The Journal of neuroscience: the official journal of the Society for Neuroscience. 2000;20: 7517–24. doi: 10.1523/JNEUROSCI.20-20-07517.2000 11027209 PMC6772866

[66] de la Cruz IP, Levin JZ, Cummins C, Anderson P, Horvitz HR. *sup-9*, *sup-10*, and *unc-93* may encode components of a two-pore K+ channel that coordinates muscle contraction in *Caenorhabditis elegans*. The Journal of neuroscience: the official journal of the Society for Neuroscience. 2003;23: 9133–45. 14534247 10.1523/JNEUROSCI.23-27-09133.2003 PMC6740817

[67] de la Cruz IP, Ma L, Horvitz HR. The *Caenorhabditis elegans* iodotyrosine deiodinase ortholog SUP-18 functions through a conserved channel SC-box to regulate the muscle two-pore domain potassium channel SUP-9. PLoS genetics. 2014;10: e1004175. doi: 10.1371/journal.pgen.1004175 24586202 PMC3930498

[68] Levin JZ, Horvitz HR. Three new classes of mutations in the *Caenorhabditis elegans* muscle gene *sup-9*. Genetics. 1993;135: 53–70. doi: 10.1093/genetics/135.1.53 8224828 PMC1205627

[69] Trent C, Tsuing N, Horvitz HR. Egg-laying defective mutants of the nematode *Caenorhabditis elegans*. Genetics. 1983;104: 619–47. doi: 10.1093/genetics/104.4.619 11813735 PMC1202130

[70] Yemini E, Jucikas T, Grundy LJ, Brown AEX, Schafer WR. A database of *C*. *elegans* behavioral phenotypes. Nat Methods. 2013;10: 877–879. doi: 10.1038/nmeth.2560 23852451 PMC3962822

[71] Aryal P, Abd-Wahab F, Bucci G, Sansom MSP, Tucker SJ. A hydrophobic barrier deep within the inner pore of the TWIK-1 K2P potassium channel. Nat Commun. 2014;5: 4377. doi: 10.1038/ncomms5377 25001086 PMC4102122

[72] Aryal P, Sansom MSP, Tucker SJ. Hydrophobic gating in ion channels. J Mol Biol. 2015;427: 121–130. doi: 10.1016/j.jmb.2014.07.030 25106689 PMC4817205

[73] Cho YS, Chen C-H, Hu C, Long J, Ong RTH, Sim X, et al. Meta-analysis of genome-wide association studies identifies 8 new loci for type 2 diabetes in East Asians. Nat Genet. 2011;44: 67–72. doi: 10.1038/ng.1019 22158537 PMC3582398

[74] Vierra NC, Dadi PK, Jeong I, Dickerson M, Powell DR, Jacobson DA. Type 2 Diabetes-Associated K+ Channel TALK-1 Modulates β-Cell Electrical Excitability, Second-Phase Insulin Secretion, and Glucose Homeostasis. Diabetes. 2015;64: 3818–3828. doi: 10.2337/db15-0280 26239056 PMC4613978

[75] Vierra NC, Dadi PK, Milian SC, Dickerson MT, Jordan KL, Gilon P, et al. TALK-1 channels control β cell endoplasmic reticulum Ca2+ homeostasis. Sci Signal. 2017;10. doi: 10.1126/scisignal.aan2883 28928238 PMC5672804

[76] Chapman CG, Meadows HJ, Godden RJ, Campbell DA, Duckworth M, Kelsell RE, et al. Cloning, localisation and functional expression of a novel human, cerebellum specific, two pore domain potassium channel. Brain Res Mol Brain Res. 2000;82: 74–83. doi: 10.1016/s0169-328x(00)00183-2 11042359

[77] Vega-Saenz de Miera E, Lau DH, Zhadina M, Pountney D, Coetzee WA, Rudy B. KT3.2 and KT3.3, two novel human two-pore K(+) channels closely related to TASK-1. J Neurophysiol. 2001;86: 130–142. doi: 10.1152/jn.2001.86.1.130 11431495

[78] Kasap M, Dwyer DS. Na+ leak-current channel (NALCN) at the junction of motor and neuropsychiatric symptoms in Parkinson’s disease. J Neural Transm (Vienna). 2021;128: 749–762. doi: 10.1007/s00702-021-02348-6 33961117

[79] Barel O, Shalev SA, Ofir R, Cohen A, Zlotogora J, Shorer Z, et al. Maternally inherited Birk Barel mental retardation dysmorphism syndrome caused by a mutation in the genomically imprinted potassium channel KCNK9. Am J Hum Genet. 2008;83: 193–199. doi: 10.1016/j.ajhg.2008.07.010 18678320 PMC2495061

[80] Graham JM, Zadeh N, Kelley M, Tan ES, Liew W, Tan V, et al. KCNK9 imprinting syndrome-further delineation of a possible treatable disorder. Am J Med Genet A. 2016;170: 2632–2637. doi: 10.1002/ajmg.a.37740 27151206

[81] Meng J, Ahamed T, Yu B, Hung W, Mouridi SE, Leclercq-Blondel A, et al. A descending interneuron with depolarized resting membrane potential controls *C. elegans* motor states. bioRxiv; 2022. p. 2022.04.06.487231. doi: 10.1101/2022.04.06.487231

[82] Yue Z, Zhang Y, Yu B, Xu Y, Chen L, Li Y, et al. K2P channel TWK-40 Regulates a Rhythmic Behavior in *C. elegans*. bioRxiv; 2022. p. 2022.04.09.487752. doi: 10.1101/2022.04.09.487752

[83] Davis MW, Hammarlund M, Harrach T, Hullett P, Olsen S, Jorgensen EM. Rapid single nucleotide polymorphism mapping in *C*. *elegans*. BMC genomics. 2005;6: 118. doi: 10.1186/1471-2164-6-118 16156901 PMC1242227

[84] Mello CC, Kramer JM, Stinchcomb D, Ambros V. Efficient gene transfer in *C*.*elegans*: extrachromosomal maintenance and integration of transforming sequences. The EMBO journal. 1991;10: 3959–70. 1935914 10.1002/j.1460-2075.1991.tb04966.x PMC453137

[85] Frokjaer-Jensen C, Davis MW, Hopkins CE, Newman BJ, Thummel JM, Olesen SP, et al. Single-copy insertion of transgenes in *Caenorhabditis elegans*. Nature genetics. 2008;40: 1375–83. doi: 10.1038/ng.248 18953339 PMC2749959

[86] Shen Z, Zhang X, Chai Y, Zhu Z, Yi P, Feng G, et al. Conditional knockouts generated by engineered CRISPR-Cas9 endonuclease reveal the roles of coronin in *C*. *elegans* neural development. Dev Cell. 2014;30: 625–636. doi: 10.1016/j.devcel.2014.07.017 25155554

[87] Farboud B, Meyer BJ. Dramatic enhancement of genome editing by CRISPR/Cas9 through improved guide RNA design. Genetics. 2015;199: 959–71. doi: 10.1534/genetics.115.175166 25695951 PMC4391549

[88] Kumar S, Stecher G, Li M, Knyaz C, Tamura K. MEGA X: Molecular Evolutionary Genetics Analysis across Computing Platforms. Mol Biol Evol. 2018;35: 1547–1549. doi: 10.1093/molbev/msy096 29722887 PMC5967553

