# Supplementary figures and images for: Differential modulation of C. elegans motor behavior by NALCN and two-pore domain potassium channels
Source: PLoS Genet. 2022 Apr 28;18(4):e1010126. doi: 10.1371/journal.pgen.1010126 (PMC9049526; doi:10.1371/journal.pgen.1010126)

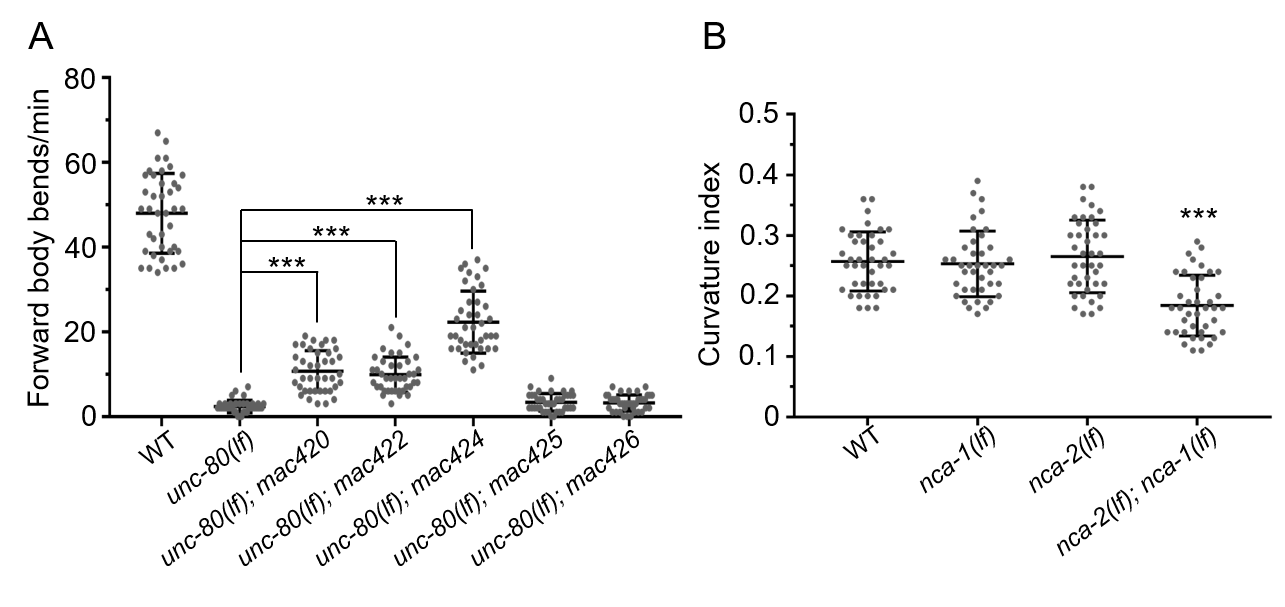

Supplement: S1 Fig — (A) Touch-triggered forward locomotion of unc-80(lf); sup mutants. mac420, mac422 and mac424 could significantly improve the defective locomotion of unc-80(lf) mutants. (B) Curvature indices of nca-1(lf) single, nca-2(lf) single and nca-2(lf); nca-1(lf) double mutants. 40 animals were quantified for each genotype. Statistics: Bonferroni multiple comparison with one-way ANOVA. ***, p < 0.001. (TIF) [file pgen.1010126.s001.tif]

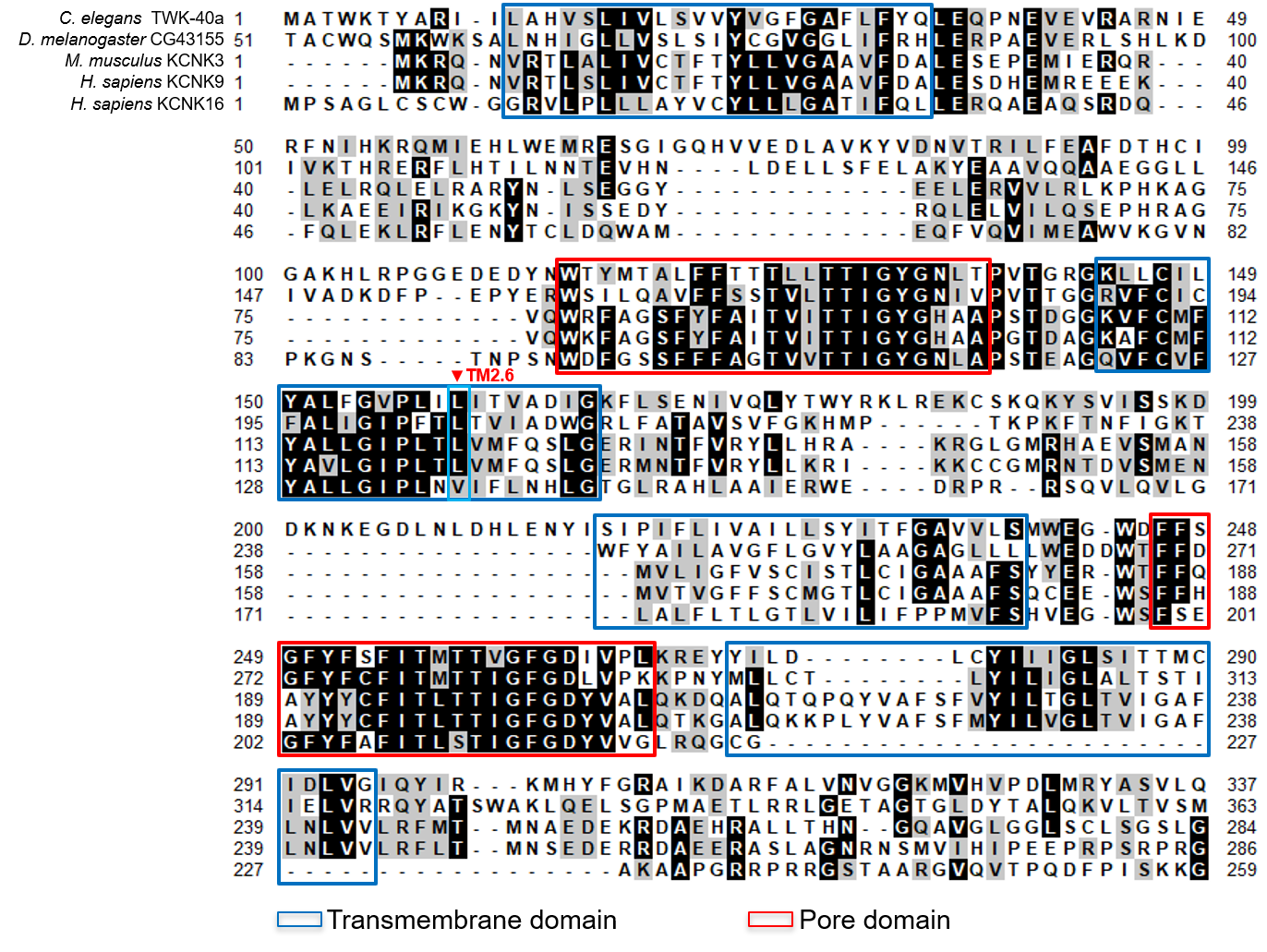

Supplement: S2 Fig — The four transmembrane domains are enclosed in blue boxes, and the two pore domains are in red boxes. The amino acid at TM2.6 is indicated with a red arrowhead. The first 50 amino acids of the Drosophila CG43155 were omitted. C-terminal regions of TWK-40a (aa 338–393), Drosophila CG43155 (aa 364–411), M. musculus KCNK3 (aa 285–409), H. sapiens KCNK9 (aa 287–401) and H. sapiens KCNK16 (aa 260–262) were not included in the alignment. (TIF) [file pgen.1010126.s002.tif]

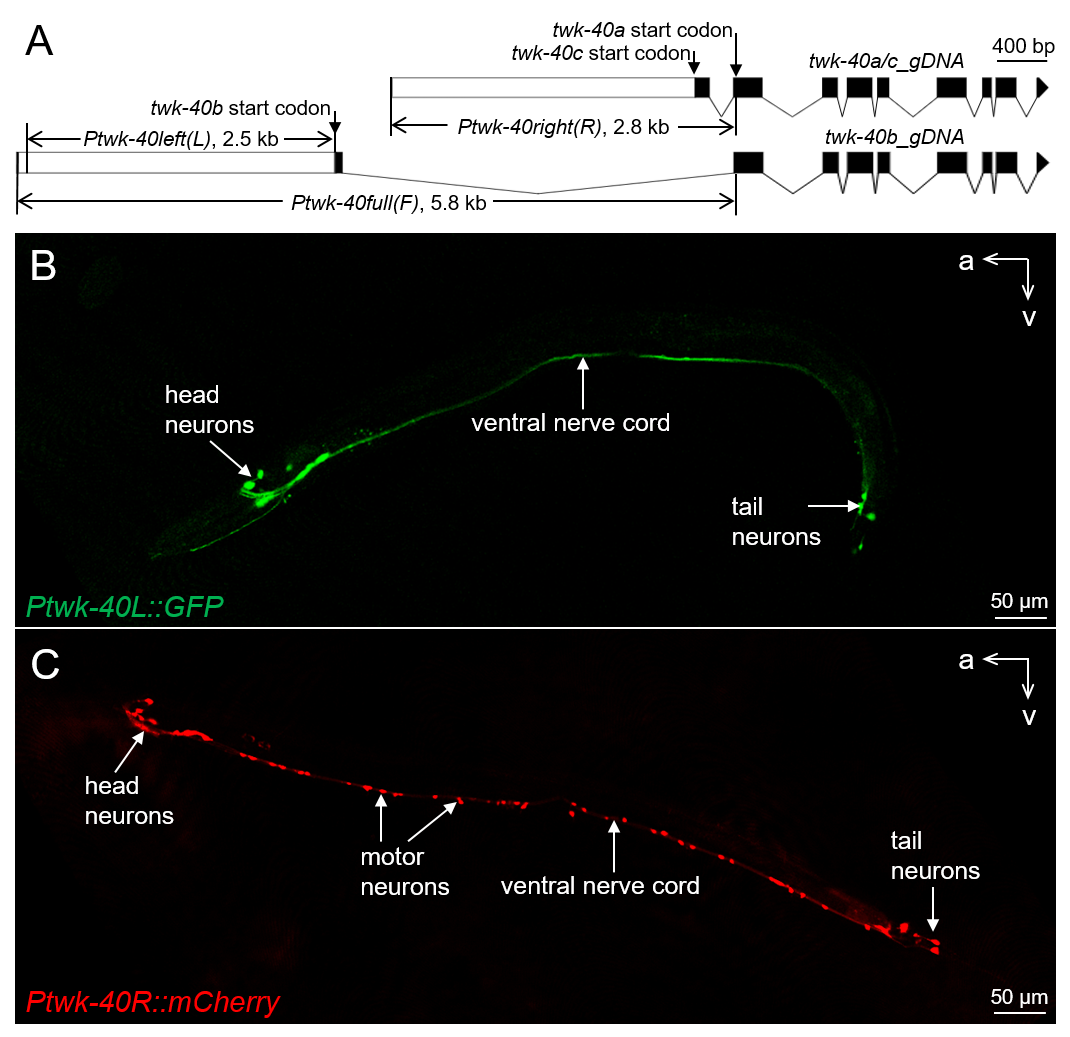

Supplement: S3 Fig — (A) The genomic positions of Ptwk-40F, Ptwk-40L and Ptwk-40R promoters. (B) GFP driven by the Ptwk-40L promoter labeled some head neurons, ventral nerve cord and tail neurons. Ventral cord motor neurons were not obviously labeled. (C) mCherry driven by the Ptwk-40R promoter labeled multiple head neurons, ventral nerve cord, ventral motor neurons and tail neurons. a: anterior; v: ventral. (TIF) [file pgen.1010126.s003.tif]

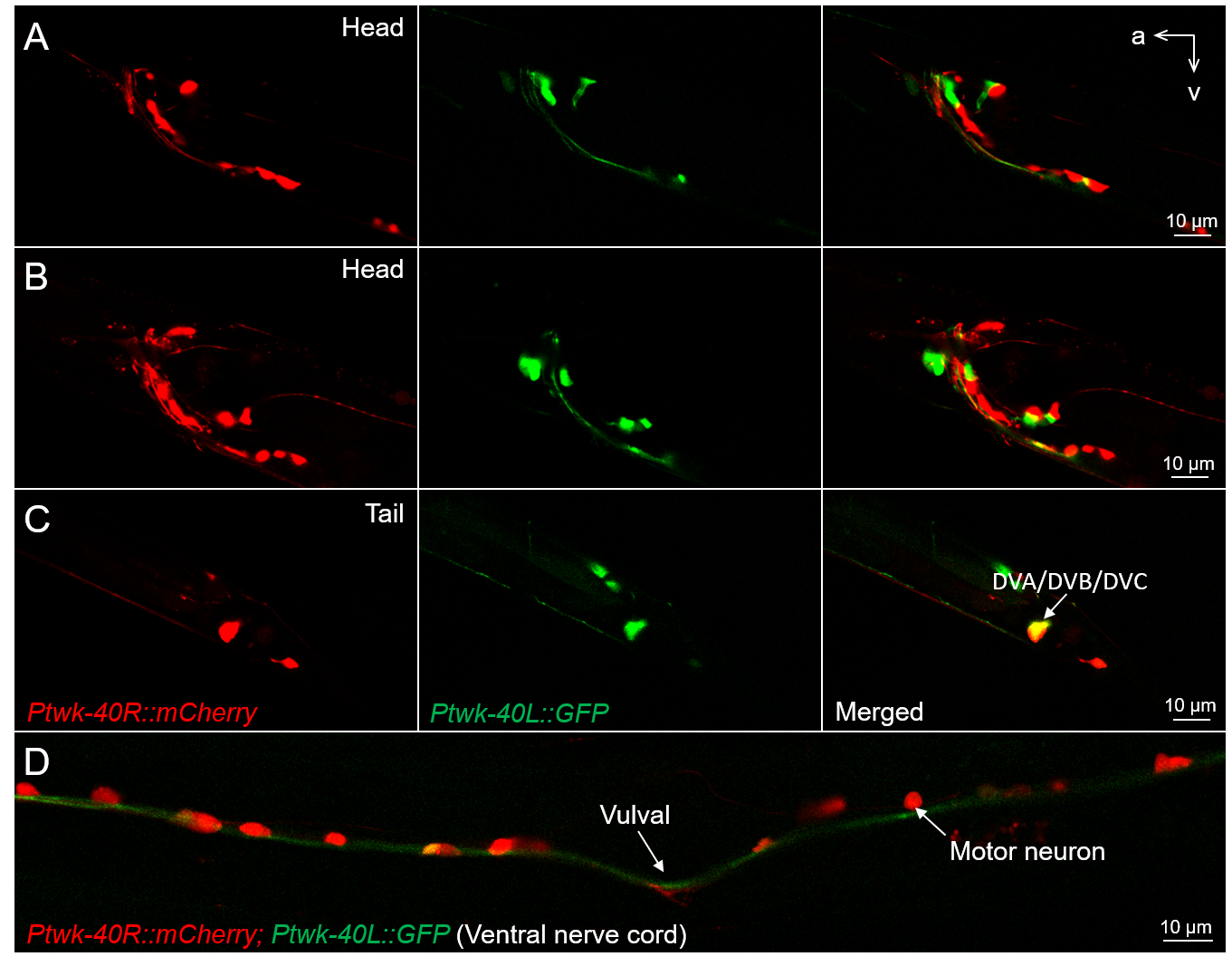

Supplement: S4 Fig — (A) Confocal pictures of head neurons labeled by Ptwk-40R::mCherry (left panel) and Ptwk-40L::GFP (middle panel). The merged picture was shown on the right. (B) Confocal pictures of the same animal as in (A) at a different focal plane. (C) A tail neuron(s) at the position of DVA/DVB/DVC neurons was co-labeled by mCherry and GFP. (D) Ventral nerve cord in the middle region of an animal showing the mCherry-labeled motor neurons and GFP-labeled cord. For all pictures, a: anterior; v: ventral. (TIF) [file pgen.1010126.s004.tif]

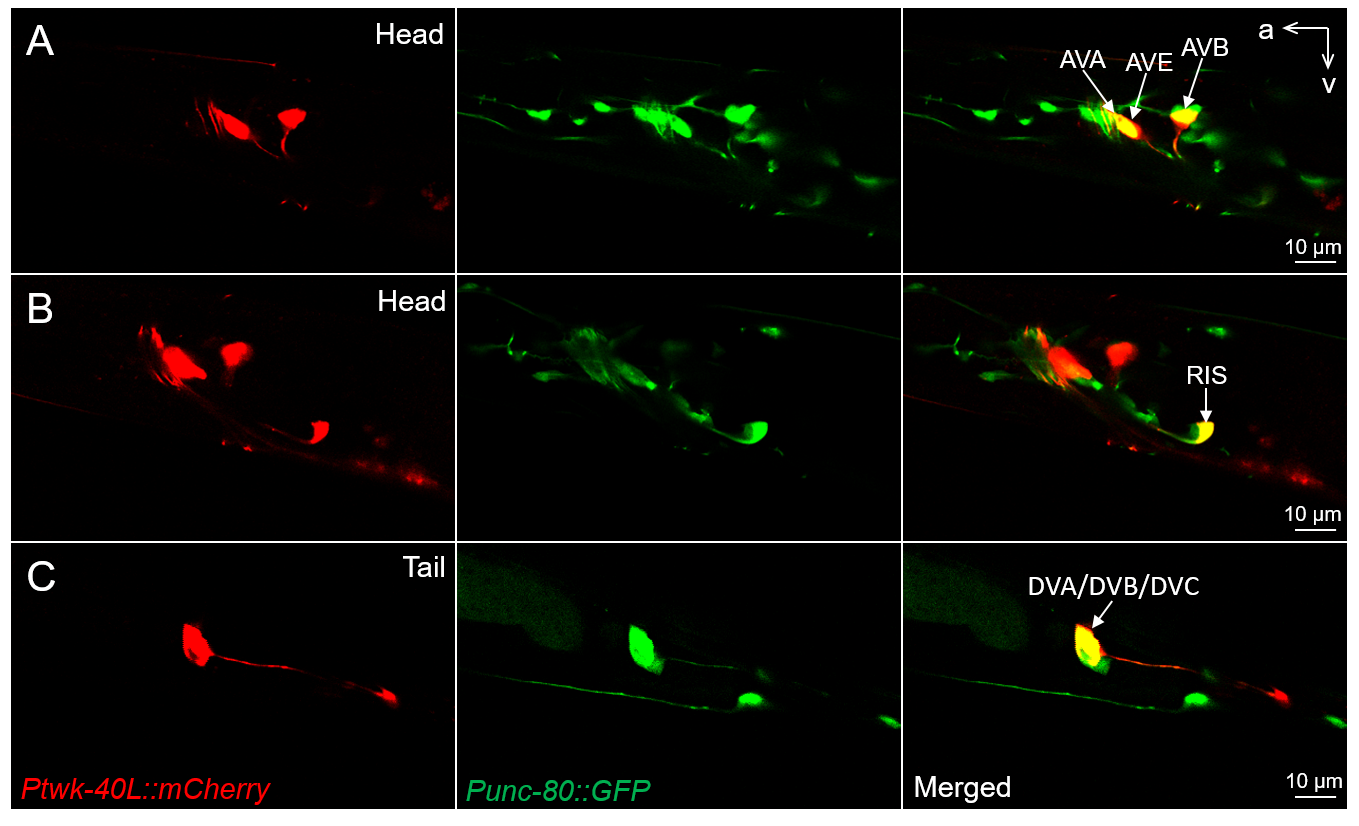

Supplement: S5 Fig — (A) Confocal pictures of adult head neurons expressing the Ptwk-40L::mCherry transgene (left panel) and the Punc-80::GFP transgene (middle panel). (B) Confocal picture of the same animal as in (A) on a different focal plane. (C) A tail neuron(s) at the position of DVA/DVB/DVC was co-labeled by mCherry (left panel) and GFP (middle panel). The merged pictures were shown on the right. For all pictures, a: anterior; v: ventral. (TIF) [file pgen.1010126.s005.tif]

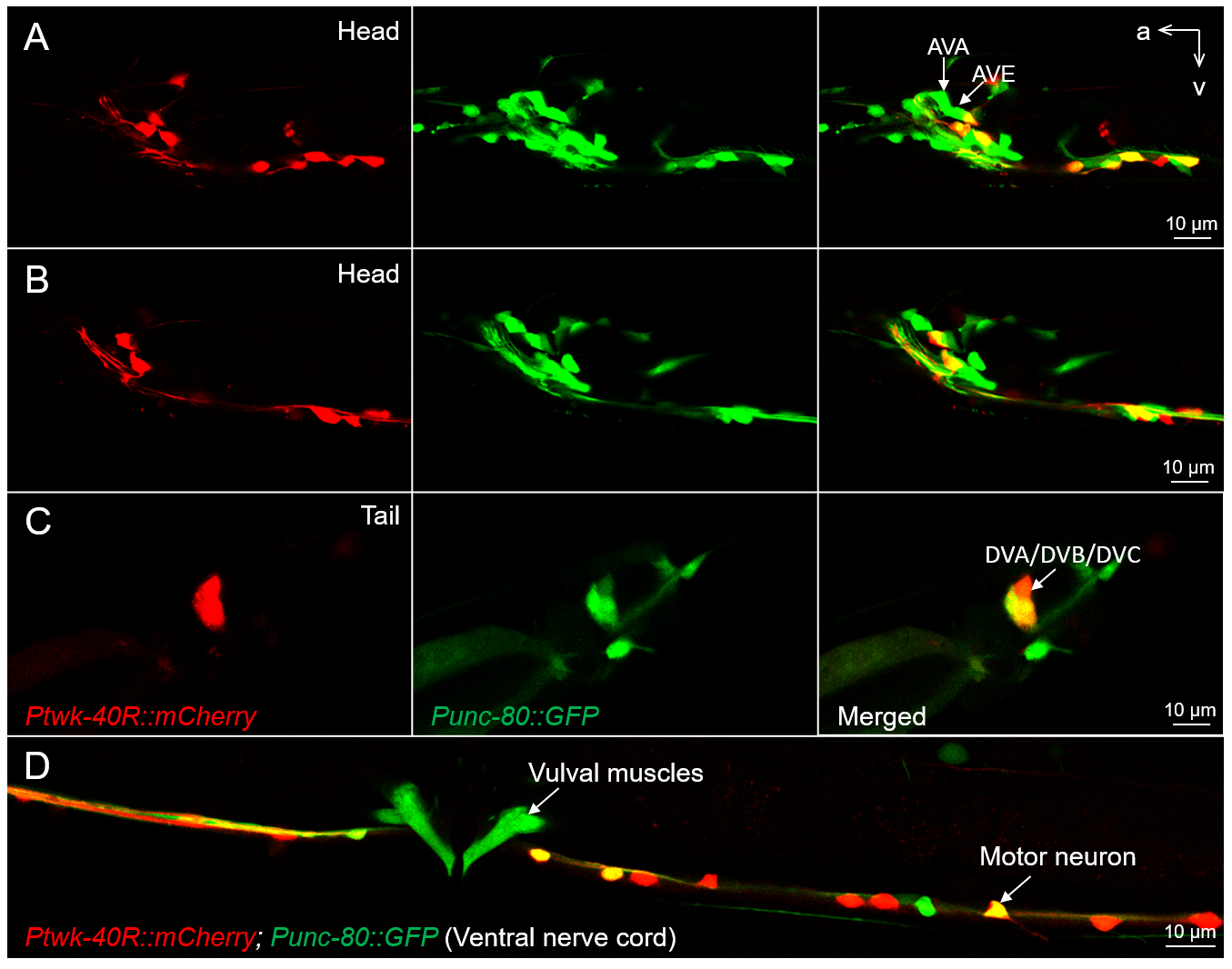

Supplement: S6 Fig — (A) Confocal pictures of adult head neurons expressing the Ptwk-40R::mCherry transgene (left panel) and the Punc-80::GFP transgene (middle panel). The merged picture was shown on the right. (B) Confocal picture of the same animal as in (A) at a different focal plane. (C) A tail neuron(s) at the position of DVA/DVB/DVC appeared to be co-labeled by mCherry (left panel) and GFP (middle panel). (D) Ventral cord in the middle region of an animal showing several motor neurons co-labeled by mCherry and GFP. For all pictures, a: anterior; v: ventral. (TIF) [file pgen.1010126.s006.tif]

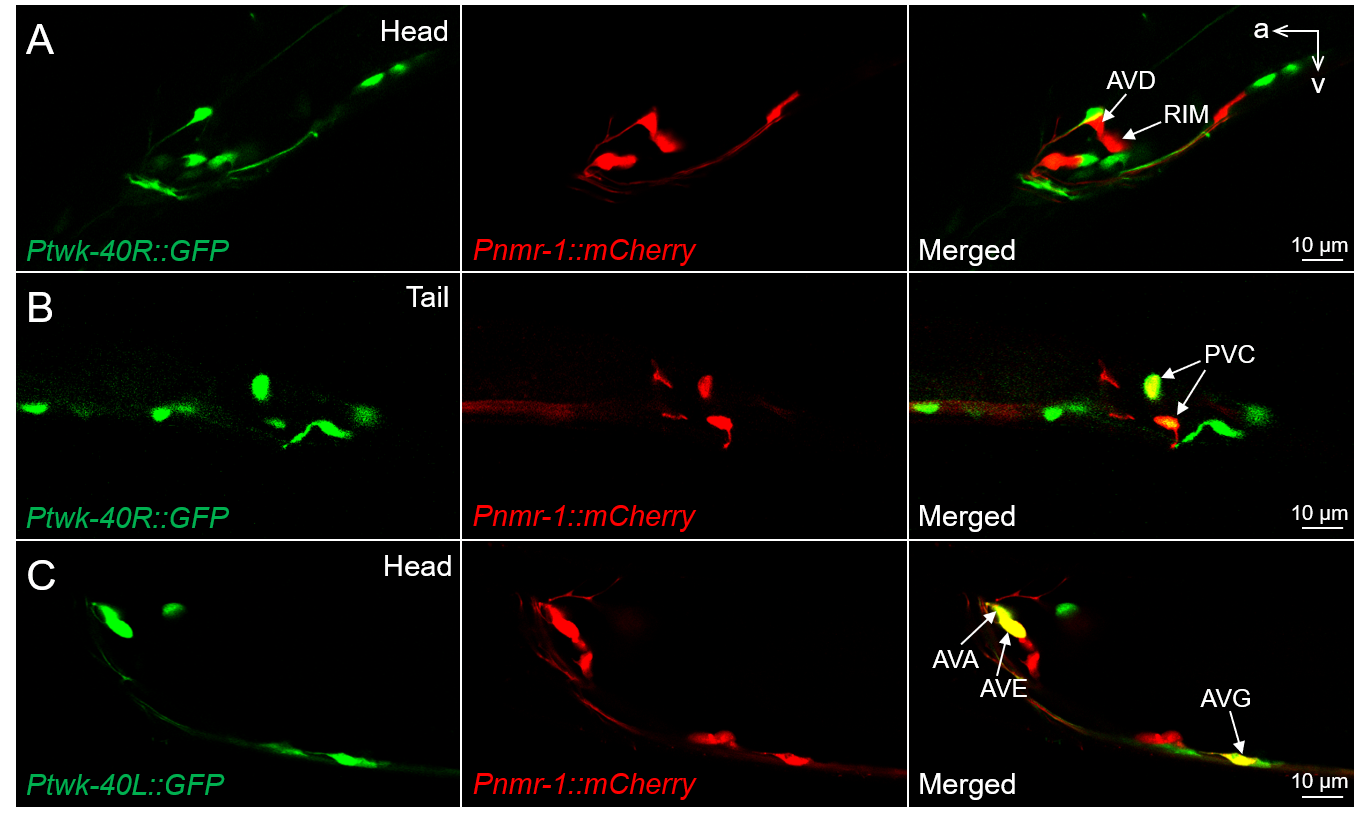

Supplement: S7 Fig — (A) Confocal pictures of adult head neurons expressing the Ptwk-40R::GFP transgene (left panel) and the Pnmr-1::mCherry transgene (middle panel). The merged picture was shown on the right. (B) Confocal pictures of adult tail neurons expressing the Ptwk-40R::GFP transgene (left panel) and the Pnmr-1::mCherry transgene (middle panel). (C) Confocal pictures of adult head neurons expressing the Ptwk-40L::GFP transgene (left panel) and the Pnmr-1::mCherry transgene (middle panel). For all pictures, a: anterior; v: ventral. (TIF) [file pgen.1010126.s007.tif]

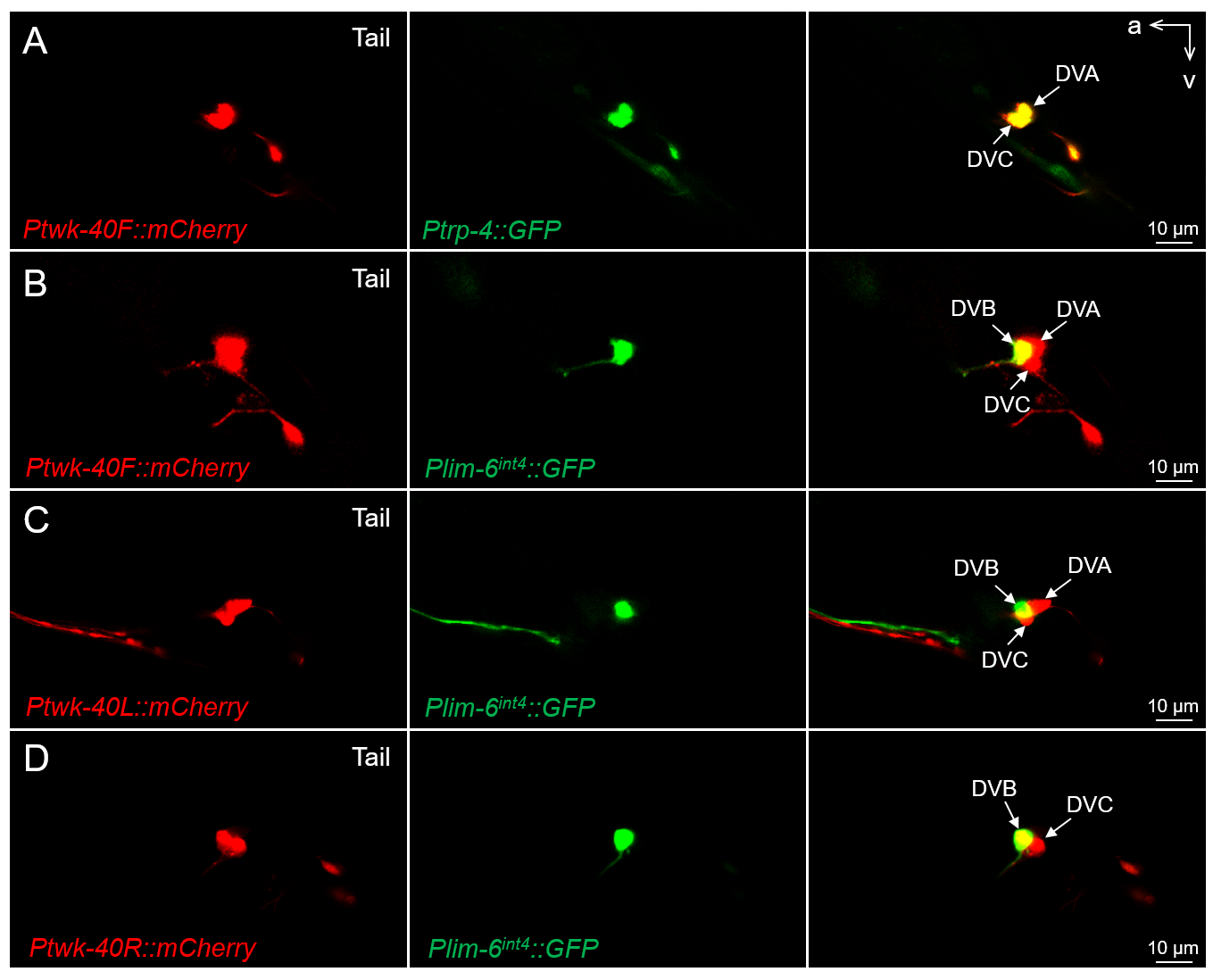

Supplement: S8 Fig — (A) Confocal pictures of adult tail neurons expressing the Ptwk-40F::mCherry transgene (left panel) and the Ptrp-4::GFP transgene (middle panel). (B) Confocal pictures of adult tail neurons expressing the Ptwk-40F::mCherry transgene (left panel) and the Plim-6int4::GFP transgene (middle panel). (C) Confocal pictures of adult tail neurons expressing the Ptwk-40L::mCherry transgene (left panel) and the Plim-6int4::GFP transgene (middle panel). (D) Confocal pictures of adult tail neurons expressing the Ptwk-40R::mCherry transgene (left panel) and the Plim-6int4::GFP transgene (middle panel). The merged pictures were shown on the right. For all pictures, a: anterior; v: ventral. (TIF) [file pgen.1010126.s008.tif]

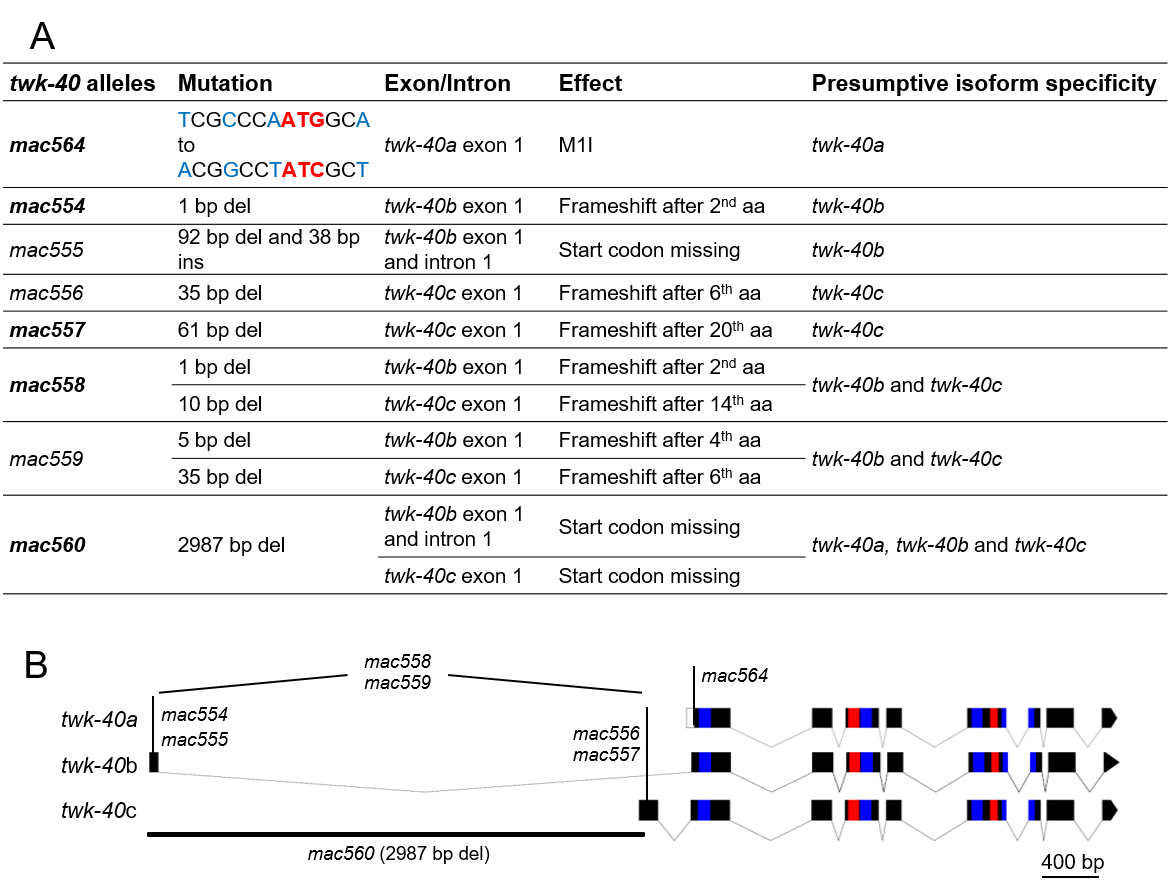

Supplement: S9 Fig — (A) List of presumptive twk-40 isoform-specific mutations. (B) Genomic positions of twk-40 isoform-specific mutations. (TIF) [file pgen.1010126.s009.tif]

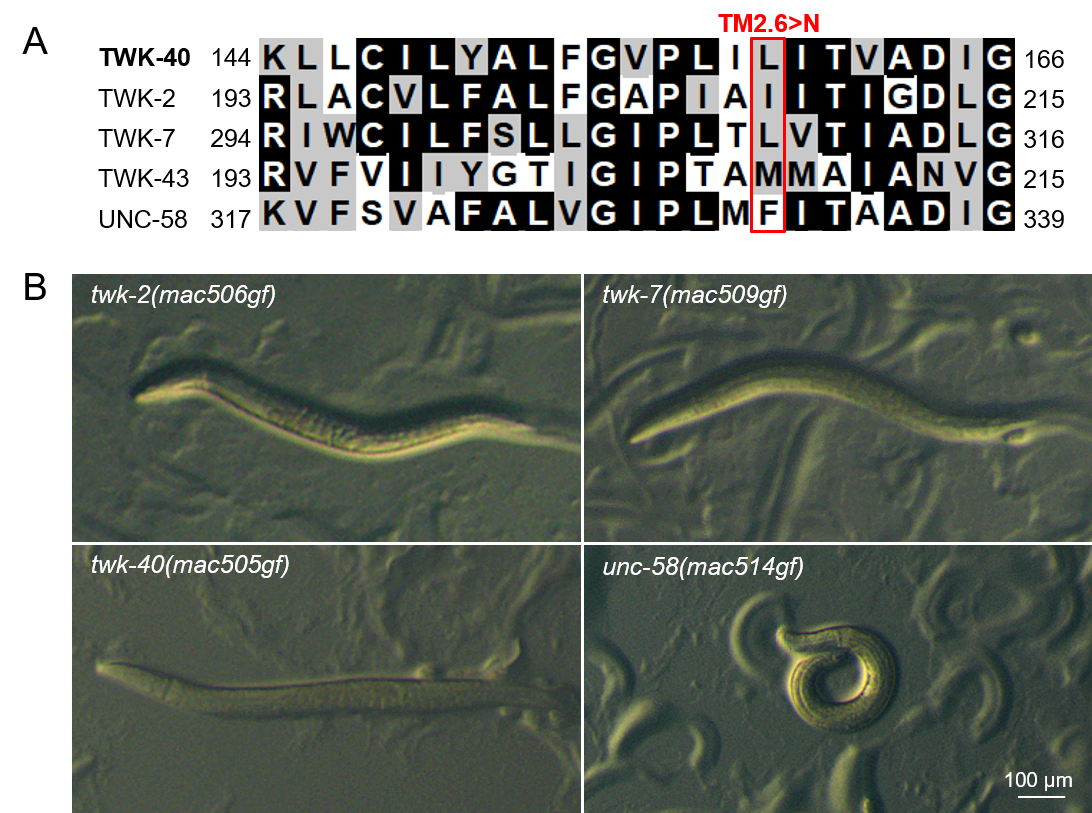

Supplement: S10 Fig — (A) Alignment of the 2nd transmembrane domains of indicated TWK channels. TM2.6 was substituted with asparagine (N). (B) Representative pictures of twk(gf) mutants. (TIF) [file pgen.1010126.s010.tif]

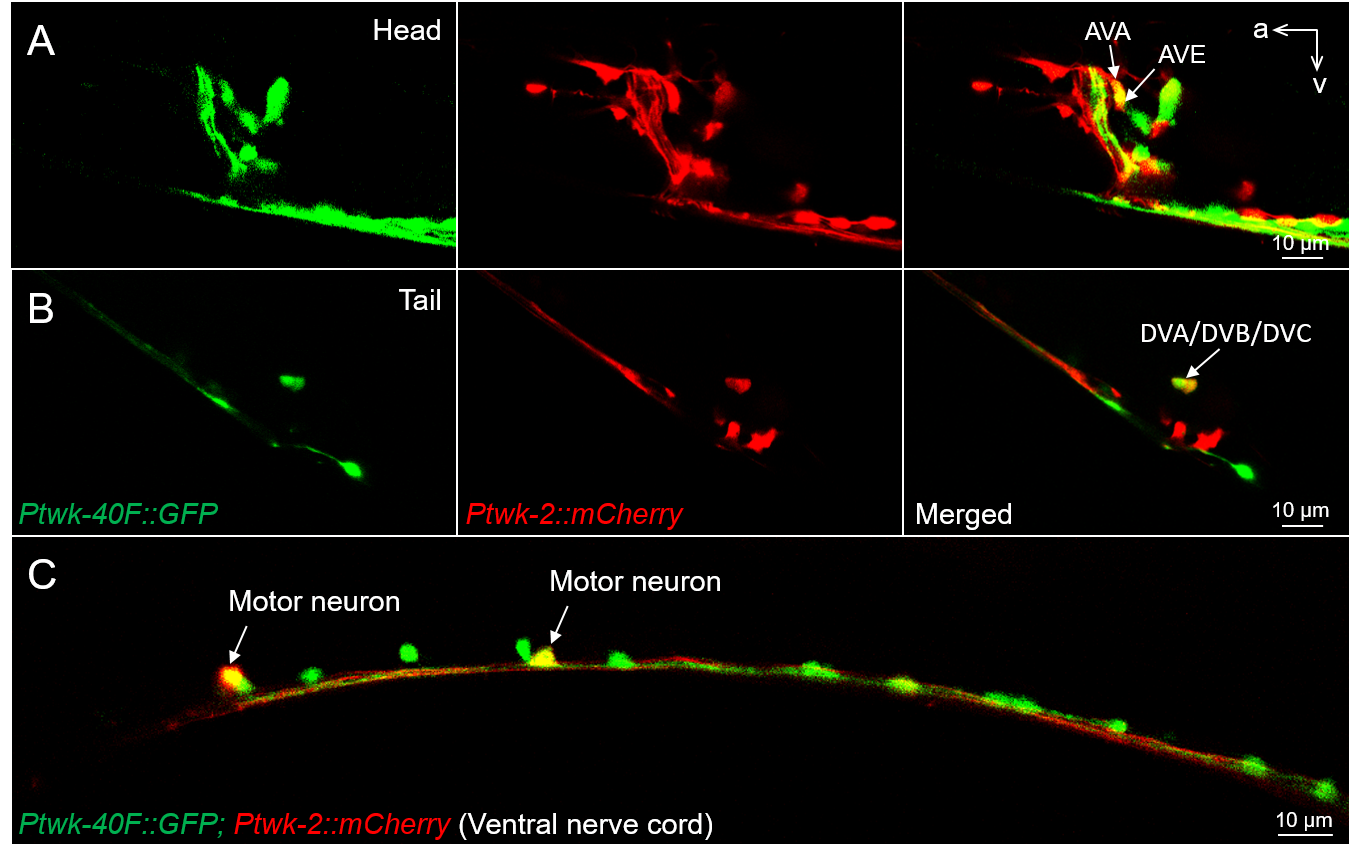

Supplement: S11 Fig — (A) Confocal pictures of adult head neurons expressing the Ptwk-40F::GFP transgene (left panel) and a Ptwk-2::mCherry transgene (middle panel). (B) An unidentified tail neuron(s) at the position of DVA/DVB/DVC was co-labeled by GFP (left panel) and mCherry (middle panel). The merged pictures were shown on the right. (C) Ventral cord in the middle region of a transgenic animal showing two motor neurons co-labeled by GFP and mCherry. For all pictures, a: anterior; v: ventral. (TIF) [file pgen.1010126.s011.tif]

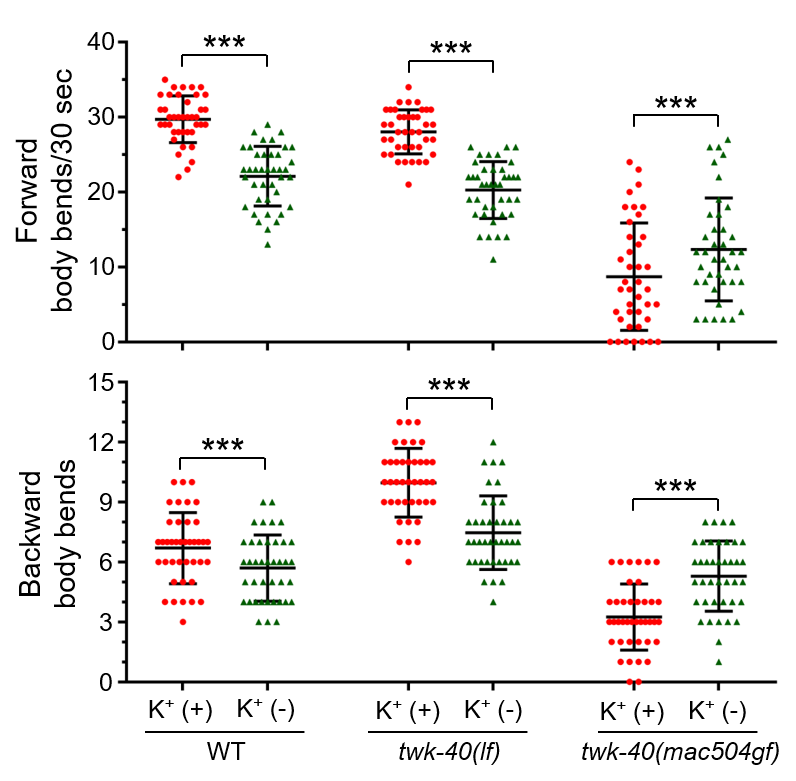

Supplement: S12 Fig — 40 animals were quantified for each assay condition. Statistics: two-tailed unpaired Student’s t-test. ***, p < 0.001. (TIF) [file pgen.1010126.s012.tif]
