# Supplementary material for: Differential modulation of C. elegans motor behavior by NALCN and two-pore domain potassium channels
Source: PLoS Genet. 2022 Apr 28;18(4):e1010126. doi: 10.1371/journal.pgen.1010126 (PMC9049526; doi:10.1371/journal.pgen.1010126)
Supplement: S1 Text — (DOCX) [file pgen.1010126.s043.docx]

**Strains and plasmid constructions**

*C. elegans* strains were grown at 20°C with OP50 bacteria as food. N2 (Bristol) is the reference wild type strain. Strains used in this study include:

CB4856 (Hawaiian) [1]

CSM659 *unc-80(mac379lf) V* (reference loss-of-function allele, lf) [2]

CSM740 *unc-80(mac379lf) V; mac420*

CSM742 *unc-80(mac379lf) V; mac422*

CSM757 *unc-80(mac379lf) V; mac424*

CSM758 *twk-40(mac425lf) III; unc-80(mac379lf) V* (backcrossed 6 times)

CSM759 *twk-40(mac426lf) III; unc-80(mac379lf) V* (backcrossed 6 times)

CSM1117 *twk-40(mac425lf) III* (reference loss-of-function allele, lf)

CSM1169 *twk-40(mac426lf) III*

CSM881 *twk-40(mac469lf) III; unc-80(mac379lf) V*

CSM884 *twk-40(mac472lf) III; unc-80(mac379lf) V*

CSM660 *unc-79(mac383lf) III* (reference loss-of-function allele, lf) [2]

CSM662 *nca-2(gk5lf) III; nca-1(gk9lf) IV* (reference loss-of-function alleles, lf) [3,4]

VC9 *nca-2(gk5lf) III* (reference loss-of-function alleles, lf) [3,4]

VC12 *nca-1(gk9lf) IV* (reference loss-of-function alleles, lf) [3,4]

CSM720 *nlf-1(mac409lf) X* (reference loss-of-function allele, lf) [2]

CSM1025 *unc-79(mac383lf) III twk-40(mac425lf) III*

CSM1027 *nca-2(gk5lf) III twk-40(mac425lf) III; nca-1(gk9lf) IV*

CSM1029 *twk-40(mac425lf) III; nlf-1(mac409lf) X*

DR1089 *nca-1(e625gf) IV* (reference gain-of-function allele, gf) [4]

CSM1276 *twk-40(mac504gf) III* (I158-L159 deletion, gain-of-function)

CSM1277 *twk-40(mac505gf) III* (L159N, gain-of-function)

CSM1278 *twk-40(mac504gf) III; nca-1(e625gf) IV*

CSM1279 *twk-40(mac505gf) III; nca-1(e625gf) IV*

CSM1280 *twk-40(mac504gf) III; unc-80(mac379lf) V*

CSM1281 *twk-40(mac505gf) III; unc-80(mac379lf) V*

CSM1319 *twk-2(mac506gf) II* (reference gain-of-function allele, gf)

CSM1320 *twk-2(mac507lf) II* (reference loss-of-function allele, lf)

CSM1321 *twk-2(mac508) II* (deletion/frameshift)

CSM1322 *twk-7(mac509gf) III* (reference gain-of-function allele, gf)

CSM1323 *twk-7(mac510lf) III* (reference loss-of-function allele, lf)

CSM1324 *twk-7(mac511) III* (deletion/frameshift)

CSM1325 *twk-17(mac512lf) X* (reference loss-of-function allele, lf)

CSM1326 *twk-17(mac513) X* (7 aa in-frame deletion)

CSM1327 *unc-58(mac514gf) X* (reference gain-of-function allele, gf)

CSM1328 *unc-58(mac515lf) X* (reference loss-of-function allele, lf)

CSM1329 *unc-58(mac516) X* (deletion/frameshift)

CSM1330 *twk-43(mac517) V* (M208N)

CSM1331 *twk-43(mac518lf) V* (reference loss-of-function allele, lf)

CSM1332 *twk-43(mac519) V* (19 aa in-frame deletion)

CSM1333 *twk-3(mac520) II* (deletion/frameshift)

CSM1334 *twk-13(mac521lf) V* (reference loss-of-function allele, lf)

CSM1335 *twk-13(mac522) V* (deletion/frameshift)

CSM1336 *twk-46(mac523lf) V* (reference loss-of-function allele, lf)

CSM1337 *twk-46(mac524) V* (deletion/frameshift)

CSM1338 *twk-2(mac507lf) II; twk-40(mac425lf) III*

CSM1339 *twk-7(mac510lf) III twk-40(mac425lf) III*

CSM1340 *twk-40(mac425lf) III; twk-17(mac512lf) X*

CSM1341 *twk-40(mac425lf) III; unc-58(mac515lf) X*

CSM1342 *twk-2(mac507lf) II; twk-40(mac505gf) III*

CSM1343 *twk-7(mac510lf) III twk-40(mac505gf) III*

CSM1344 *twk-40(mac505gf) III; twk-17(mac512lf) X*

CSM1345 *twk-40(mac505gf) III; unc-58(mac515lf) X*

CSM1346 *twk-2(mac506gf) II; twk-40(mac425lf) III*

CSM1347 *twk-7(mac509gf) III twk-40(mac425lf) III*

CSM1348 *twk-40(mac425lf) III; unc-58(mac514gf) X*

CSM1349 *twk-2(mac507lf) II; unc-80(mac379lf) V*

CSM1350 *twk-7(mac510lf) III; unc-80(mac379lf) V*

CSM1351 *unc-80(mac379lf) V; twk-17(mac512lf) X*CSM1352 *unc-80(mac379lf) V; unc-58(mac515lf) X*

CSM1353 *twk-2(mac506gf) II; nca-1(e625gf) IV*

CSM1354 *twk-7(mac509gf) III; nca-1(e625gf) IV*

CSM1355 *nca-1(e625gf) IV; unc-58(mac514gf) X*

CSM1356 *twk-26(mac525lf) IV* (reference loss-of-function allele, lf)

CSM1357 *twk-26(mac526) IV* (deletion/frameshift)

CSM1358 *twk-29(mac527lf) I* (reference loss-of-function allele, lf)

CSM1359 *twk-29(mac528) I* (deletion/frameshift)

CSM1360 *twk-30(mac529lf) I* (reference loss-of-function allele, lf)

CSM1361 *twk-30(mac530) I* (deletion/frameshift)

CSM1362 *twk-35(mac531lf) V* (reference loss-of-function allele, lf)

CSM1363 *twk-35(mac532) V* (deletion/insertion/frameshift)

CSM1364 *twk-48(mac533lf) III* (reference loss-of-function allele, lf)

CSM1365 *twk-48(mac534) III* (deletion/frameshift)

CSM1366 *twk-49(mac535lf) II* (reference loss-of-function allele, lf)

CSM1367 *twk-49(mac536) II* (deletion/frameshift)

CSM1368 *twk-40(mac425lf) III; twk-13(mac521lf) V*

CSM1369 *twk-40(mac425lf) III; twk-26(mac525lf) IV*

CSM1370 *twk-29(mac527lf) I; twk-40(mac425lf) III*

CSM1371 *twk-30(mac529lf) I; twk-40(mac425lf) III*

CSM1372 *twk-40(mac425lf) III; twk-35(mac531lf) V*

CSM1373 *twk-40(mac425lf) III; twk-43(mac518lf) V*

CSM1374 *twk-40(mac425lf) III; twk-46(mac523lf) V*

CSM1375 *twk-48(mac533lf) III twk-40(mac425lf) III*

CSM1376 *twk-49(mac535lf) II; twk-40(mac425lf) III*

CSM1382 *twk-2(mac507lf) II; twk-40(mac504gf) III*

CSM1383 *twk-2(mac537)* (G181E) *II; twk-40(mac505gf) III*

CSM1405 *twk-40(mac554) III*

CSM1406 *twk-40(mac555) III*

CSM1407 *twk-40(mac556) III*

CSM1408 *twk-40(mac557) III*

CSM1409 *twk-40(mac558) III*

CSM1410 *twk-40(mac559) III*

CSM1411 *twk-40(mac560) III*

CSM1416 *twk-40(mac564) III*

**Molecular biology**

The 9.5 kb *Ptwk-40L::twk-40b_gDNA* transgene, including the *Ptwk-40L* promoter (Fig S3A, 2.5 kb upstream of the *twk-40b* start codon) and the full-length *twk-40b* coding region, was amplified using *C. elegans* genomic DNAs as template. The 5.3 kb *Ptwk-40R::twk-40a/c_gDNA,* including the *Ptwk-40R* promoter (Fig S3A, 2.8 kb upstream of the *twk-40a/c* start codon) and the full-length *twk-40a/c* coding region, was similarly amplified from *C. elegans* gDNAs.

Plasmid constructions were as follows:

*Ptwk-40F::GFP*: the 5.8 kb *Ptwk-40F* promoter upstream of the start codon of *twk-40a* was amplified by PCR and subcloned to pPD95_79 vector using *Sbf*I/*Xma*I restriction sites.

*Ptwk-40F::mCherry*: the *Ptwk-40F* promoter was amplified by PCR and subcloned to pPD95_79*-mCherry* vector using *Sbf*I/*Xma*I restriction sites.

*Ptwk-40L::GFP:* the 2.5 kb *Ptwk-40L* promoter upstream of the start codon of *twk-40b* was amplified by PCR and subcloned to pPD95_79 vector using *Pst*I/*Xma*I restriction sites.

*Ptwk-40L::mCherry*: the *Ptwk-40L* promoter was amplified by PCR and subcloned to pPD95_79*-mCherry* vector using *Pst*I/*Xma*I restriction sites.

*Ptwk-40R::GFP*: the 2.8 kb *Ptwk-40R* promoter upstream of the start codon of *twk-40a* was amplified by PCR and subcloned to pPD95_79 vector using *Pst*I/*Xma*I restriction sites.

*Ptwk-40R::mCherry*: the 2.8 kb *Ptwk-40R* promoter was amplified by PCR and subcloned to pPD95_79*-mCherry* vector using *PstI*/*Xma*I restriction sites.

*Ptwk-2::GFP*: a 3.1 kb *twk-2* promoter upstream of the start codon of *twk-2a* was amplified by PCR and subcloned to pPD95_79 vector using *Sbf*I/*Xma*I restriction sites.

*Ptrp-4::GFP*: a *trp-4* promoter (4.2 kb upstream of the *trp-4* start codon) was amplified by PCR and subcloned to pPD95_79 using In-fusion HD Cloning Kit (Clontech).

*Plim-6^int4^::GFP*: a 291 bp fragment of the *lim-6* fourth intron was amplified by PCR and subcloned to pPD95_79 vector using *Sbf*I/*Xma*I restriction sites.

*Ptwk-40F::twk-40a_cDNA*: a full-length *twk-40a* cDNA was amplified from *C. elegans* cDNAs and subcloned to the pPD95_79-*Ptwk-40F* vector using *Xma*I restriction site.

*Ptwk-40L::twk-40b_*cDNA, *Ptwk-40R::twk-40b_cDNA, Punc-119::twk-40b_cDNA, Punc-80::twk-40b_cDNA, Pnlf-1::twk-40b_cDNA or Pnmr-1::twk-40b_cDNA*: the full-length *twk-40b* cDNA was amplified from *C. elegans* cDNAs and subcloned to the pPD95_79-*Ptwk-40L,* pPD95_79-*Ptwk-40R,* pPD95_79-*Punc-119,* pPD95_79-*Punc-80,* pPD95_79-*Pnlf-1 or* pPD95_79-*Pnmr-1* vector using *Xma*I restriction site.

*Ptwk-40L::Cas9::NLS::3’UTR*: the *Ptwk-40L* promoter was amplified and subcloned to the pPD95_79-*Punc-80::Cas9::NLS::3’UTR* vector by replacing *Punc-80* using *Sph*I/*Xma*I restriction sites*.*

*Ptwk-40L::twk-40a_cDNA, Ptwk-40R::twk-40a_cDNA or Pnmr-1::twk-40a_cDNA*: a *twk-40a* cDNA was amplified and subcloned to the pPD95_79-*Ptwk-40L,* pPD95_79-*Ptwk-40R or* pPD95_79-*Pnmr-1* vector using *Xma*I restriction site.

*Pacr-2::twk-40a_cDNA*: an *acr-2* promoter (3.4 kb upstream of the *acr-2* start codon) was amplified and subcloned to pPD95_79 using *Sbf*I*/XmaI* restriction sites. The *twk-40a* cDNA was amplified and subcloned to the pPD95_79-*Pacr-2* vector using *Xma*I restriction site.

*Pcfi-1::twk-40a_cDNA*: a *cfi-1* promoter (4.9 kb upstream of the *cfi-1* start codon) was amplified and subcloned to pPD95_79 using *Sbf*I*/XmaI* restriction sites. The *twk-40a* cDNA was amplified and subcloned to the pPD95_79-*Pcfi-1* vector using *Xma*I restriction site.

*Ptrp-4::twk-40a_cDNA*: the *twk-40a* cDNA was amplified and subcloned to the pPD95_79-*Ptrp-4* vector using In-fusion HD Cloning Kit.

*Plim-6^int4^::twk-40a_cDNA*: the *twk-40a* cDNA was amplified and subcloned to the pPD95_79-*Plim-6^int4^* vector using *Xma*I restriction site.

*Ptwk-40F::twk-40a(mac504gf)_cDNA*: a *Ptwk-40F::twk-40a(mac504gf)_cDNA* linear fragment was amplified using pPD95_79-*Ptwk-40F::twk-40a_cDNA* plasmid as template and re-ligated using In-fusion HD Cloning Kit. PCR primers were designed to generate the *mac504* mutation from wildtype *twk-40a* template.

*Ptwk-40F::twk-40a(I158del)_cDNA or Ptwk-40F::twk-40a(L159del)_cDNA*: the *Ptwk-40F::twk-40a(I158del)_cDNA or* the *Ptwk-40F::twk-40a(L159del)_cDNA* linear fragment was amplified using pPD95_79-*Ptwk-40F::twk-40a_cDNA* plasmid as template and re-ligated using In-fusion HD Cloning Kit. PCR primers were designed to generate the I158del or L159del mutation from wildtype *twk-40a* template.

*Ptwk-40F::twk-40a(I158-L159del, T161I)_cDNA, Ptwk-40F::twk-40a(I158-L159del, T161N)_cDNA, or Ptwk-40F::twk-40a(I158-L159del, T161K)_cDNA*: the *Ptwk-40F::twk-40a(I158-L159del, T161I)_cDNA, Ptwk-40F::twk-40a(I158-L159del, T161N)_cDNA or Ptwk-40F::twk-40a(I158-L159del, T161K)_cDNA* linear fragment was amplified using pPD95_79-*Ptwk-40F:: twk-40a(mac504gf)_cDNA* plasmid as template and re-ligated using In-fusion HD Cloning Kit. PCR primers were designed to generate the *(I158-L159del, T161I), (I158-L159del, T161N) or (I158-L159del, T161K)* mutation from wildtype *twk-40a* template.

*Ptwk-40F::twk-40a(mac505gf)_cDNA*: a *Ptwk-40F::twk-40a(mac505gf)_cDNA* linear fragment was amplified using pPD95_79-*Ptwk-40F::twk-40a_cDNA* plasmid as template and re-ligated using In-fusion HD Cloning Kit. PCR primers were designed to generate the *mac505* mutation from wildtype *twk-40a* template.

*Ptwk-40F::twk-40a(L159I)_cDNA, Ptwk-40F::twk-40a(L159V)_cDNA, Ptwk-40F::twk-40a(L159D)_cDNA or Ptwk-40F::twk-40a(L159K)_cDNA*: the *Ptwk-40F::twk-40a(L159I)_cDNA, Ptwk-40F::twk-40a(L159V)_cDNA, Ptwk-40F::twk-40a(L159D)_cDNA or Ptwk-40F::twk-40a(L159K)_cDNA* linear fragment was amplified using pPD95_79-*Ptwk-40F:: twk-40a_cDNA* plasmid as template and re-ligated using In-fusion HD Cloning Kit. PCR primers were designed to generate *L159I, L159V, L159D or L159K* mutation from wildtype *twk-40a* template.

*Ptwk-40L::twk-40a(mac505gf)_cDNA, Ptwk-40R::twk-40a(mac505gf)_cDNA or Pnmr-1::twk-40a(mac505gf)_*cDNA: a *twk-40a(mac505gf)_cDNA* was amplified and subcloned to the pPD95_79-*Ptwk-40L::twk-40a_cDNA,* *Ptwk-40R::twk-40a_cDNA or* *Pnmr-1::twk-40a_cDNA* vector by replacing *twk-40a cDNA* using *XmaI* restriction site.

*Punc-119::twk-2a_cDNA, Punc-119::twk-7_cDNA, Punc-119::twk-17a_cDNA, Punc-119::twk-30_gDNA, Punc-119::twk-48_cDNA or Punc-119::unc-58a_cDNA*: the full-length *twk-2a* cDNA, *twk-7 cDNA,* *twk-17a* cDNA, *twk-30 gDNA,* *twk-48* cDNA or *unc-58a* cDNA was amplified and subcloned to the pPD95_79-*Punc-119* vector using *Xma*I/*Kpn*l restriction site.

The cDNA inserts of all constructs were verified by sequencing. PCR primers are listed in S8 Table.

**References**

1. Wicks SR, Yeh RT, Gish WR, Waterston RH, Plasterk RH. Rapid gene mapping in *Caenorhabditis elegans* using a high density polymorphism map. Nature genetics. 2001;28: 160–4. doi:10.1038/88878

2. Zhou C, Luo J, He X, Zhou Q, He Y, Wang X, et al. The NALCN Channel Regulator UNC-80 Functions in a Subset of Interneurons To Regulate *Caenorhabditis elegans* Reversal Behavior. G3 (Bethesda). 2020;10: 199–210. doi:10.1534/g3.119.400692

3. Jospin M, Watanabe S, Joshi D, Young S, Hamming K, Thacker C, et al. UNC-80 and the NCA ion channels contribute to endocytosis defects in synaptojanin mutants. Current biology : CB. 2007;17: 1595–600. doi:10.1016/j.cub.2007.08.036

4. Yeh E, Ng S, Zhang M, Bouhours M, Wang Y, Wang M, et al. A putative cation channel, NCA-1, and a novel protein, UNC-80, transmit neuronal activity in *C. elegans*. PLoS biology. 2008;6: e55. doi:10.1371/journal.pbio.0060055
